# Bacterial, archaeal and micro-eukaryotic communities characterize a disease-suppressive or conducive soil and a cultivar resistant or susceptible to common scab

**DOI:** 10.1038/s41598-019-51570-6

**Published:** 2019-10-16

**Authors:** Jan Kopecky, Zuzana Samkova, Ensyeh Sarikhani, Martina Kyselková, Marek Omelka, Vaclav Kristufek, Jiri Divis, Geneviève G. Grundmann, Yvan Moënne-Loccoz, Marketa Sagova-Mareckova

**Affiliations:** 1 Crop Research Institute, Epidemiology and Ecology of Microorganisms, Drnovská 509, 161 06 Prague 6, Czech Republic; 2 Biology Centre of the Czech Academy of Sciences, v. v. i., Institute of Soil Biology, Na Sádkách 7, 370 05 České Budějovice, Czech Republic; 3 Faculty of Mathematics and Physics, Department of Probability and Mathematical Statistics, Charles University, Sokolovská 83, 186 75 Prague 8, Czech Republic; 4 Faculty of Agriculture, University of South Bohemia, Studentská 13, 370 05 České Budějovice, Czech Republic; 5 Univ Lyon, Université Claude Bernard Lyon 1, CNRS, INRA, VetAgro Sup, UMR5557 Ecologie Microbienne, F-69622 Villeurbanne, France; 6 Department of Microbiology, Nutrition and Dietetics, Faculty of Agrobiology, Food and Natural Resources, Czech University of Life Sciences, Kamycka 129, Prague 6, Czech Republic

**Keywords:** Microbial ecology, Microbial ecology

## Abstract

Control of common scab disease can be reached by resistant cultivars or suppressive soils. Both mechanisms are likely to translate into particular potato microbiome profiles, but the relative importance of each is not known. Here, microbiomes of bulk and tuberosphere soil and of potato periderm were studied in one resistant and one susceptible cultivar grown in a conducive and a suppressive field. Disease severity was suppressed similarly by both means yet, the copy numbers of *txtB* gene (coding for a pathogenicity determinant) were similar in both soils but higher in periderms of the susceptible cultivar from conducive soil. Illumina sequencing of 16S rRNA genes for bacteria (completed by 16S rRNA microarray approach) and archaea, and of 18S rRNA genes for micro-eukarytes showed that in bacteria, the more important was the effect of cultivar and diversity decreased from resistant cultivar to bulk soil to susceptible cultivar. The major changes occurred in proportions of *Actinobacteria, Chloroflexi*, and *Proteobacteria*. In archaea and micro-eukaryotes, differences were primarily due to the suppressive and conducive soil. The effect of soil suppressiveness × cultivar resistance depended on the microbial community considered, but differed also with respect to soil and plant nutrient contents particularly in N, S and Fe.

## Introduction

Suppressive soils are described as soils in which disease severity remains low, in spite of the presence of a pathogen, a susceptible host, and climatic conditions favorable for disease development^1,2^. Relatively few soils with suppressive character have been described in the world to date^3^ although it is of prime interest to understand and conserve their functioning because they may help us to learn how to establish suppressive character of soils at other sites^4^. Common scab (CS) of potatoes is a soil-borne disease caused by *Streptomyces* spp. that produce thaxtomin phytotoxins, and for which suppressive soils were reported mostly in the USA^5,6^. In these systems, disease control is largely attributed to biological interactions (mostly competition and antagonism) between plant-beneficial microbiota and pathogens mediated via antibiotic production or enzymatic activities^4,7^. In particular, nonpathogenic *Streptomyces* spp. were correlated with CS suppressiveness^6,7^, and it was also hypothesized that other actinobacteria may be involved in this disease suppression^4^.

High levels of resistance to common scab are not found in most commercially significant cultivars of potato^8^. The resistance to CS is manifested by different quantities of pathogenic streptomycetes in their tubers but not in roots or rhizosphere^9^. Yet, potato cultivars differing in resistance to common scab also have different ecophysiologies as they differ in chemical composition of the potato periderm and preferences in nutrient utilization^10^. Since various bacterial communities are associated with either resistant or susceptible cultivars^9^, interactions between potato plants with different genotypes and associated microbial communities may further influence the disease development under specific soil conditions.

In our previous investigations, CS suppressiveness was studied in two areas (Vyklantice and Zdirec) from the Czech Republic, in field trials^11,12^ and pot experiments^13^. We found that the suppressive character of the fields differed between the two locations, because it was attributed to soil chemical characteristics in the Zdirec area, versus microbial community interactions in the Vyklantice area^11^. Therefore, this work we aimed at disentangling the relative effects of soil suppressiveness and potato resistance on the structure of microbial communities in the soil in contact with potato tubers (i.e. tuberosphere, also termed geocauloshere), using suppressive and conducive soils from the Vyklantice area since biotic interactions were determined as responsible for soil supressiveness there.

Often, the focus in suppressive soil assessment has been put on bacteria^7,14^. Yet, fungi can be also important for crop protection^3^ and the role of micro-eukaryotes and their participation in top-down control has been typically neglected^15^ although many of them can be relevant to soil suppressiveness because microfauna and mesofauna members may consume pathogens, increase nutrient turnover or maintain specific diversity by feeding on the dominant bacterial taxa^16,17^. Archaea also are part of the rhizosphere microbiome. whether they can participate in biocontrol interactions remains unknown^18,19^, hence the importance of including them in microbial assessments. Finally, CS-susceptible and resistant potato cultivars have not been compared yet in terms of their respective interactions with the soil microbial community in CS suppressive soils. Our objective was to test whether both suppressive soil and resistant cultivar represent significant ecological factors shaping microbial communities of the potato tuberosphere. To this end, we used a field experiment that included a combination of (i) disease suppressive vs conducive soils, and (ii) resistant vs susceptible cultivars. The study compared spatial compartments of tuberosphere, potato periderm and bulk soil because it was determined that only in tuberosphere differences between factors influencing CS severity occur^14,20^. Bacterial, archaeal and micro-eukaryote communities in soil and potato tuberosphere were assessed by Illumina sequencing. Above that 16S rRNA taxonomic microarray was used for its semi-quantitative approach in bacterial community assessment^21,22^ and also because our taxonomic microarray focuses on bacterial taxa possessing plant growth-promoting and antagonistic traits in soil environments^23,24^. For this study, our microarray was extended with probes focusing on CS pathogens. The results were considered in relation to CS severity observed on tuber surface, quantity of thaxtomin biosynthetic genes *txtB*, quantities of total bacteria and more specifically of actinobacteria, but also against chemical characteristics of soil and potato periderm. That was done in order to identify interactions between potato plants, microbial community and soil characteristics in common scab manifestation.

## Results

### Common scab severity and quantities of thaxtomin biosynthetic genes

**Figure 1:**
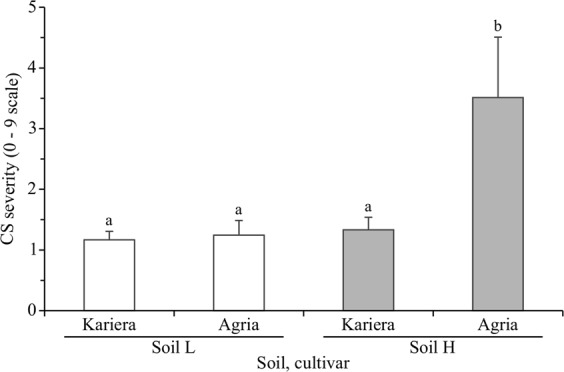
Severity of common scab of susceptible cultivar Agria and resistant cultivar Kariera in suppressive (L, low severity) and conducive (H, high severity) soils (means ± standard deviations, n = 4). Statistical significance between treatments (ANOVA) are shown with letters a and b.

In conducive soil H, severity of CS (resulting from natural field infestation) was significantly higher in susceptible cultivar Agria than resistant cultivar Kariera (Fig. 1; ANOVA, p < 0.001). In suppressive soil L, CS severity did not differ between the cultivars, and was as low as for the resistant cultivar in conducive soil. The number of *txtB* gene copies was similar in both soils (Tables S1A and S3A), while in periderm it was significantly higher (p = 0.006) in conducive than suppressive soil (Tables S1B and S3B). The two cultivars grown in the same soil had comparable quantities of *txtB* gene copies in their periderm. In summary, CS control required resistant cultivar (independently of the soil) or suppressive soil (for susceptible cultivar).

### Chemical composition of tuberosphere soil and periderm

In tuberosphere, contents of N, C, P, Ca, and soil pH were significantly higher in conducive than suppressive soil (ANOVA; all p < 0.001), while S content was significantly higher in suppressive soil (ANOVA; p < 0.001). Ca content was significantly higher in bulk soil than in tuberosphere of both soils (ANOVA; p < 0.001; Tables S2A and S3A). In periderm, N content was significantly higher in both cultivars from suppressive soil (ANOVA; p < 0.001), Ca content was significantly higher in susceptible cultivar Agria in both soils (ANOVA; p = 0.011), and Mg content was significantly higher in resistant cultivar Kariera in both soils (ANOVA; p < 0.001). Fe content was significantly higher in tuberospheres of both cultivars in conducive soil (Supplementary Table S2A), but it was highest in periderm of the resistant cultivar in suppressive soil (Supplementary Tables S2B). Fe content was affected by both field and field x cultivar interaction in both soil and periderm (ANOVA; p = 0.035; ANOVA; p = 0.006 resp. Supplementary Tables S3A,B).

In summary, (i) lower content of N, C, P, and Ca and higher content of S were found in the suppressive soil (ii) higher content of Mg, P or Fe were found in the resistant cultivar. In addition, S and Fe contents were significantly higher in tuberosphere for the combination of suppressive soil × resistant cultivar, showing an interaction effect.

### Quantities of total bacteria and actinobacteria

In tuberosphere, the quantities of bacteria (ANOVA; p < 0.001) and more specifically of actinobacteria (p = 0.006) were higher in conducive than in suppressive soil. In suppressive soil, the quantity of both bacteria (p = 0.011) and actinobacteria (p = 0.019) was significantly lower in plant tuberosphere compared to bulk soil (Tables S1A and S3A). In periderm, the quantity of actinobacteria (ANOVA; p = 0.021) was significantly higher in conducive than in suppressive soil, and was also significantly higher in susceptible cultivar Agria than in Kariera in conducive soil (Tables S1B and S3B). In summary, quantities of total bacteria and actinobacteria depended on soil (suppressive vs conducive) × cultivar (resistant vs susceptible) × compartment (periderm vs tuberosphere vs bulk soil) combination, with a trend for lower number (s) in suppressive soil and resistant cultivar.

### Bacterial community composition in bulk soil and tuberosphere by microarray analysis

The 16S rRNA taxonomic microarray previously validated for bacterial community analysis of rhizosphere soil samples^23,25^ was expanded for coverage of the genus *Streptomyces*, including pathogen species *S. scabies* and relatives (Table 1).

**Table 1:** Coverage of the probes added to the 16S rRNA microarray.

Probe	Probe sequence (5′-3′)	Coverage of *Streptomyces*		Ref.
		genus [%]	species	
KO 08	ACGGCTTCGCAGCTCATTGTA	28.0	—	^51^
Strepto1	CACGTGTGCAGCCCAAGACA	98.1	—	this work
Strepto2	ACGTGTGCAGCCCAAGACAT	98.1	—	this work
Strepto3	TTAGACCCCGTTTCCAGGGC	95.2	—	this work
Strepto5	GTATTAGACCCCGTTTCCAG	95.2	—	this work
Scab1	CCACACTCATCGGATGCCCG	1.7	*S. scabiei, stelliscabiei, europaeiscabiei, bottropensis, variabilis, deccanensis*	this work
Scab5	TCCACACTCATCGGATGCCC	1.7	“	this work
Scab6	TCATCGGATGCCCGAGAGTG	2.6	as “Scab1” + *S. variabilis, ipomoeae, neyagawaensis, torulosus*	this work
Scab7	ATGCCCGAGAGTGTCGTATC	1.5	*S. scabiei, stelliscabiei, variabilis, ossamyceticus, ipomoeae, neyagawaensis, torulosus*	this work
Scab8	GATGCCCGAGAGTGTCGTAT	2.2	as “Scab6”	this work
Scab9	GCTTTCCACACTCATCGGAT	1.7	as “Scab1”	this work
Scab11	GAGCTTTCCACACTCATCGG	1.7	as “Scab1”	this work

**Figure 2:**
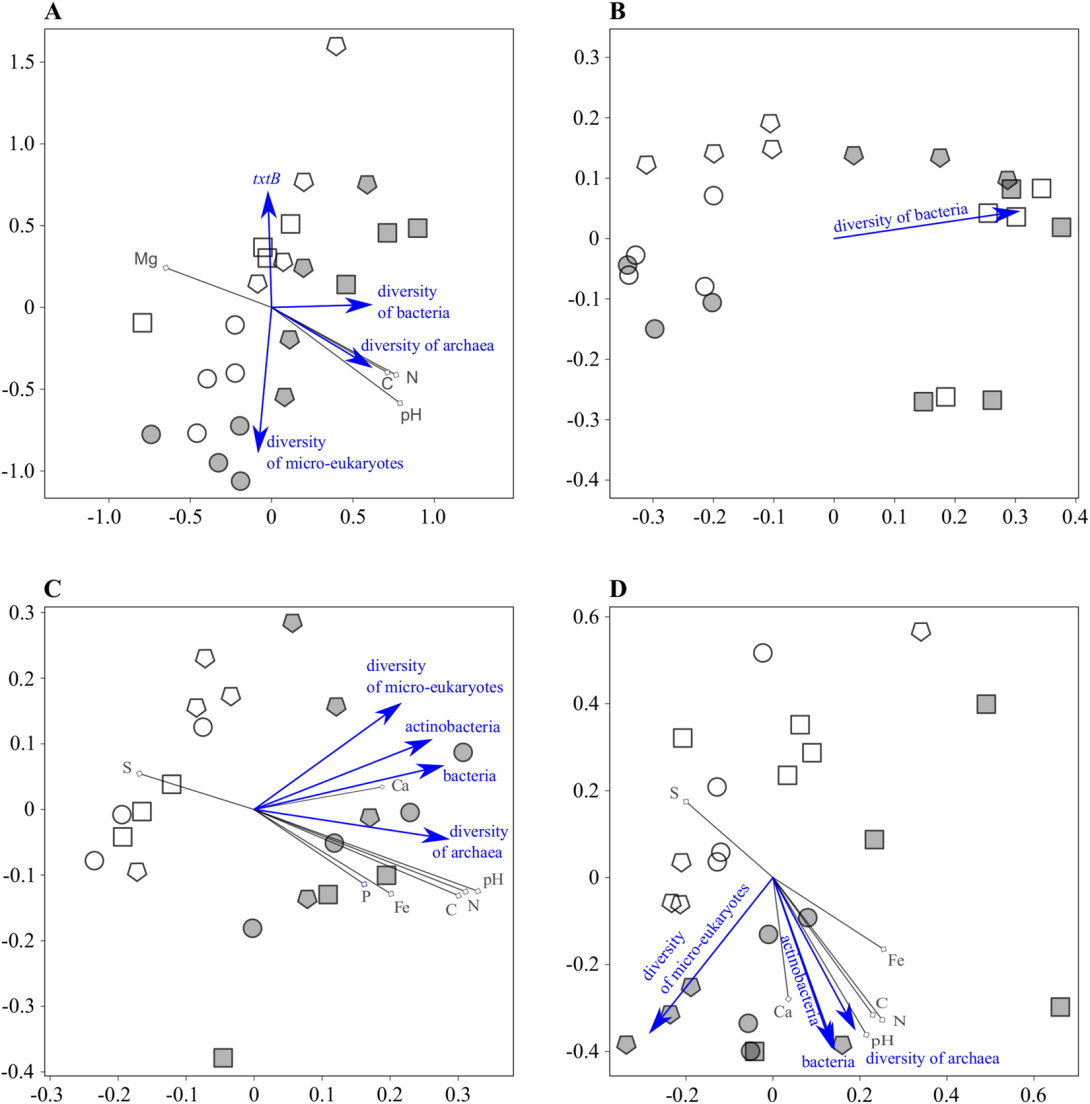
Differences in soil communities of bacteria (**A** - assayed by 16S microarray hybridization, and **B** - by 16S rRNA gene Illumina MiSeq amplicon sequencing), archaea (**C** - 16S Illumina), and eukaryotes (**D** - 18S Illumina), and the relationships to other biological and chemical characteristics of tuberosphere soil. Samples of tuberosphere (circles - susceptible potato cultivar Agria; squares - resistant cultivar Kariera), and bulk soil (pentagons) were from the fields suppressive (open symbols) and conducive (grey symbols) to the potato common scab. Non-metric multidimensional scaling of distance matrices was based on Bray-Curtis calculator with fitted vectors of environmental variables. The vector length shows the relative strengths of contributions/responses. Vectors are pointing to the same direction for positively correlated variables, and to the opposite direction for negatively correlated ones; perpendicular vectors indicate no mutual relationship.

Non-metric multidimensional scaling (NMDS) plot of sample distances calculated from microarray data demonstrated that bacterial communities in conducive and suppressive soils were distinct, and in tuberosphere they were also influenced by cultivar (Fig. 2A). According to PERMANOVA, cultivar explained 42% variability and field site 13% variability. In particular, bacterial community in tuberosphere of the susceptible cultivar was separated from those of the resistant cultivar and bulk soil. Bacterial communities were significantly closer to each other within conducive or suppressive soil when compared to all samples (PERMANOVA; p = 0.003), and samples of bacterial communities were significantly closer within each cultivar (PERMANOVA; p < 0.001) but not within each bulk soil. In tuberosphere, bacterial communities of resistant cultivar Kariera differed between the soils (PERMANOVA; p < 0.001), while bacterial communities of susceptible cultivar Agria did not differ significantly between the two soils but differed from those of resistant cultivar Kariera in each soil (PERMANOVA; p = 0.029). The permutation test identified significant relations of bacterial communities with *txtB* gene copies and Mg soil content, which were significantly higher in suppressive soil, and soil pH, C, N and diversity of bacteria, which were significantly higher in conducive soil. Diversity of micro-eukaryotes pointed to the susceptible cultivar Agria in both soils (Fig. 2A, Table S4).

In summary, analysis of bacterial community by taxonomic microarray revealed differences between both suppressive vs conducive soil and resistant vs susceptible cultivar. This was indicated by two biotic and four abiotic factors, which separated the two soils, and one biotic factor, which separated the susceptible cultivar.

### Discriminant 16S microarray probes according to soil and potato cultivar

**Figure 3:**
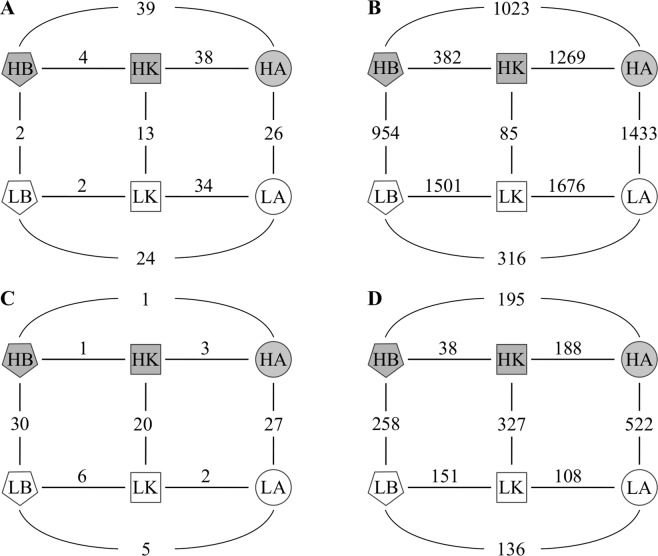
Pairwise comparisons of soil communities of bacteria (**A** - assayed by 16S microarray hybridization, and **B** - by 16S rRNA gene Illumina MiSeq amplicon sequencing), archaea (**C** - 16S Illumina), and eukaryotes (**D** - 18S Illumina) in tuberosphere of susceptible cultivar Agria (circles) and resistant cultivar Kariera (squares), and bulk soil (pentagons) from suppressive (L, open symbols) and conducive fields (H, grey symbols). Numbers indicate the probes (**A**) and OTUs (**B–D**) significantly contributing to the difference between samples in pairwise comparisons (Metastats, p < 0.05).

The Metastats analysis revealed the probes distinguishing the individual treatments in pairwise comparisons (Fig. 3A). The most pronounced differences were found between tuberospheres of the two cultivars, which were separated by signal intensities of 34 and 38 probes in suppressive and conducive soil, respectively. Only two probes discriminated between the two soils when assessing bulk soil samples, while 13 probes separated tuberospheres of the resistant cultivar Kariera, and 26 probes those of the susceptible cultivar Agria (Fig. 3A).

Considering the entire dataset including both tuberospheres of the cultivars and bulk soil, the samples from suppressive and conducive soils were significantly distinguished by the signals of 22 probes (Metastats p < 0.05). Among 13 of them with higher hybridization signals in suppressive soil, the most significantly contributing probes were Aceto3A, Acdp821, Aci1 (targeting the family *Acetobacteraceae*), PalgiG3 (*Paenibacillaceae*), Pseu33 (*Pseudomonadaceae*), Strepto5 (*Streptomycetaceae*), and Brady4 (*Bradyrhizobiaceae*). Nine of the probes were significantly higher in conducive soil, and probes Janaga 2 and 3 (*Oxalobacteraceae*) contributed most significantly to the separation of the two soils (Table S5A).

Tuberosphere samples of the cultivars were distinguished by 65 probes, 13 with higher signal in the resistant cultivar Kariera and 52 in the susceptible cultivar Agria. The probes most significantly contributing to separation of the cultivars were Strepto1, 2, and 3 (targeting the family *Streptomycetaceae*), Rzbc1247 (*Rhizobiales*), BET940 (Betaproteobacteria), and Azo5 (*Rhodospirillaceae*) with a higher signal in Kariera, and a diverse set of probes targeting *Proteobacteria* (15 probes), *Firmicutes* (2), *Planctomycetes* (2), *Actinobacteria* (2), *Bacteroidetes* (1) and *Acidobacteria* (1) with higher signal in Agria (Table S5B).

In summary, 22 probes targeting various bacterial taxa discriminated between suppressive and conducive soils, and 65 probes did between resistant and susceptible cultivars. Signals of probes targeting the CS pathogen were detected in the tuberosphere of the susceptible cultivar grown in conducive soil only.

### Bacterial community composition in bulk soil and tuberosphere by Illumina sequencing

A total of 1,213,004 16S rRNA gene sequences were obtained, out of which 944,597 (i.e. 78%) were mapped to 4001 OTUs. The number of mapped sequences per sample ranged between 31951 and 49160 with a median at 36868. On a NMDS plot, bacterial communities of resistant cultivar Kariera, susceptible cultivar Agria, and bulk soil were separated from one another within each field (Fig. 2B). The bacterial communities differed according to treatments (AMOVA, p < 0.001), with a significant difference between cultivars (AMOVA, p < 0.001) but not between suppressive and conducive soil (except when only bulk soils were compared). The permutation test identified significant influence of bacterial diversity, which pointed to the resistant cultivar Kariera (Fig. 2B, Table S4).

Both fields and cultivars were compared using significantly different OTUs (Metastats p < 0.05). The number of discriminating OTUs (Fig. 3B) was only 85 between both fields for resistant cultivar Kariera, 382 between bulk soil and resistant cultivar Kariera in conducive soil, and 316 between bulk soil and susceptible cultivar Agria in suppressive soil, whereas the other pairwise differences between treatments implicated 954–1676 discriminating OTUs.

**Figure 4:**
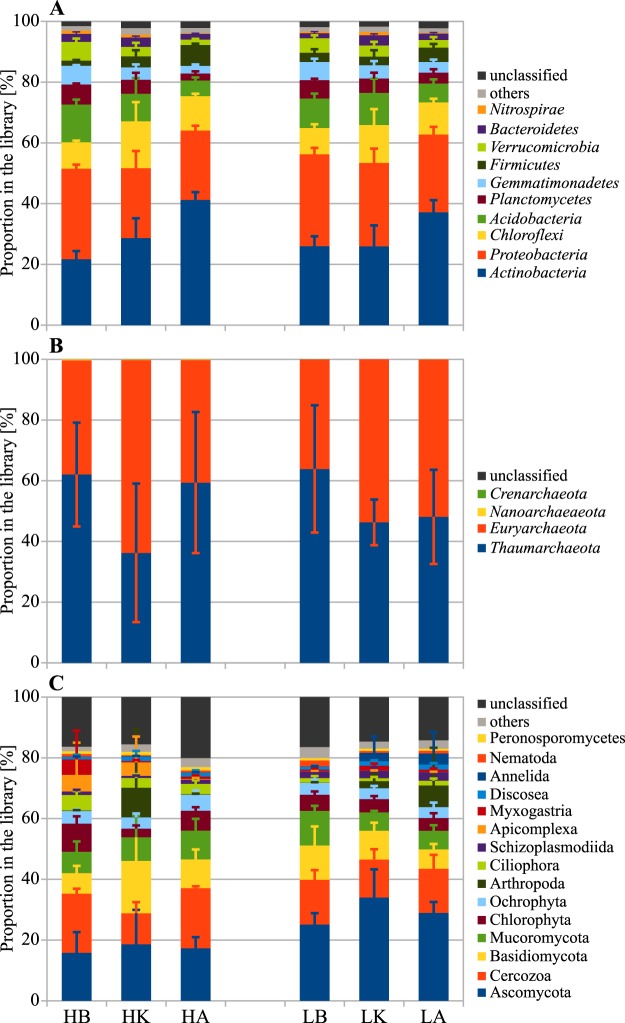
Proportions of phyla (means ± standard deviations, n = 4) in the sequence libraries of ribosomal small subunit genes from bacteria (**A**), archaea (**B**), and eukaryotes (**C**). Taxa for organisms generally larger than the sample size (*Arthropoda*, *Annelida*) were included to display the whole community although based only on shaded cells and products. Samples of tuberosphere of susceptible potato cultivar Agria (HA, LA) and resistant cultivar Kariera (HK, LK), and bulk soil (HB, LB) were from the fields suppressive (L) and conducive (H) to the potato common scab. Illumina MiSeq sequencing of amplicons were prepared with domain-specific primers.

The relative proportion of bacterial phyla did not differ between bulk soils, except that *Actinobacteria* were higher and *Acidobacteria* lower in suppressive than in conducive soil (Fig. 4A). Based on comparison with bulk soil, the tuberosphere communities implicated (i) an increase in relative proportion of *Chloroflexi* and decrease in that of *Verrucomicrobia*, *Gemmatimonadetes*, *Planctomycetes* and *Proteobacteria* in both cultivars (in the two fields; Fig. 4A), (ii) an increase in relative proportion of *Bacteroidetes* (particularly the family *Sphingobacteraceae*) in resistant cultivar Kariera (in the two fields; Fig. S1C in the Supplemental Material), and (iii) an increase in relative proportion of *Firmicutes* (especially the family *Paenibacillaceae*) and *Actinobacteria* (especially the orders *Gaiellales*, *Micrococcales*, *Frankiales* and *Streptomycetales*) in susceptible cultivar Agria (in the two fields; Fig. S1A,B). This increase in *Streptomycetales* was contributed by OTU 176, to which also the CS pathogen belongs. However, other members of this OTU contributed more significantly because this OTU was defined by centroid sequence, which was at 2.1–2.7% distance from the pathogen (Table S6B).

Rarefaction curves for bacterial communities showed that diversity did not differ between suppressive and conducive soils but differed between cultivar tuberospheres and bulk soil (two-way ANOVA, p < 0.001). The diversity was lowest in susceptible cultivar Agria, followed by bulk soil and resistant cultivar Kariera in both soils (Fig. S2A; Table S7). Significant correlative interactions (based on Spearman coefficient |ρ| ≥ 0.8) between bacterial OTUs were most numerous in bulk soil followed by resistant cultivar Kariera and susceptible cultivar Agria (Table S8).

In summary, bacteria community by Illumina sequencing revealed differences between resistant and susceptible cultivars, the latter displaying lower bacterial diversity. Differences were also found between suppressive and conducive soils, but only for bulk soil samples.

### Discriminant bacterial OTUs according to soil and potato cultivar

When considering bulk as well as tuberosphere soil samples, based on the discriminating OTUs (Metastats, p < 0.05; Fig. 4A) suppressive soil was enriched in *Plantomycetes* (OTUs 385, 2780) and *Bacteroidetes* (OTUs 1402, 1154, 1408) and conducive soil in *Actinobacteria* (OTUs 355, 1230, 886) and *Chloroflexi* (OTU 1478). Different OTUs separating the two soils were found within *Proteobacteria* (with OTUs 92, 253, 68, 592, 835 enriched in suppressive soil vs OTUs 369, 899, 2391, 1832, 2001 in conducive soil) and *Firmicutes* (OTU 3391 enriched in suppressive soil vs OTUs 2120, 2105, 1772 in conducive soil) (Table S6A).

The tuberosphere of Agria was enriched in taxa from actinobacterial orders *Frankiales* (*Frankiaceae*, *Acidothermaceae*, *Geodermatophilaceae*; OTUs 63, 20, 54, 117) and *Micrococcales* (*Intrasporangiaceae*; OTUs 13, 10) (Table S6B, Fig. S1A) and phylum *Gemmatimonadetes* (*Gemmatimonadaceae*; OTU 36), while tuberosphere of Kariera displayed significant enrichment in taxa from phylum *Acidobacteria* (OTUs 51, 275, 143, 138, 76; Table S6B, Fig. 4A). Tuberosphere communities of both cultivars were also separated by different OTUs belonging to the same taxonomic groups. These discriminating taxa included (i) *Betaproteobacteria, Burkholderiales* (OTU 38 in Agria vs OTU 199 in Kariera), (ii) *Alphaproteobacteria, Sphingomonadales* (OTU 1 in Agria vs OTUs 48, 696, 282 in Kariera), (iii) *Actinobacteria, Propionibacteriales* (OTU 138 in Agria vs OTU 6 in Kariera), *Gaiellales* (OTUs 21, 140, 41, 114, 104, 19, 8, 309, 213, 110, 23, 946 in Agria vs OTUs 16, 12, 30, 46, 107, 105, 24 in Kariera) and *Solirubrobacterales* (OTU 31 in Agria vs OTU 69 in Kariera), and (iv) *Chloroflexi* (OTUs 18, 26, 77, 137 in Agria vs OTUs 4, 55, 164, 29, 123, 284, 74 in Kariera) (Table S6B).

In summary, suppressive soil was enriched in *Plantomycetes* and *Bacteroidetes* and conducive soil in *Actinobacteria* and *Chloroflexi*, and soils also differed in their *Proteobacteria* and *Firmicutes* profiles. Resistant and susceptible cultivars differed based on 1 *Gemmatimonadetes*, 5 *Acidobacteria*, 6 *Proteobacteria*, 29 *Actinobacteria* and 11 *Chloroflexi* discriminant OTUs.

### Archaeal community composition in bulk soil and tuberosphere by Illumina sequencing, and discriminant OTUs

A total of 987,680 archaeal 16S rRNA gene sequences were obtained, out of which 545,211 (i.e. 55.2%) were mapped to 112 OTUs. The number of mapped sequences per sample ranged between 18411 and 31815 with a median at 25817. On a NMDS plot, archaeal communities were primarily separated according to conducive vs suppressive soil, though samples were more variable in conducive than suppressive soil (Fig. 2C). The archaeal communities differed overall from each other (AMOVA, p = 0.017) but while the bulk soils were significantly different (AMOVA, p = 0.006), the cultivars were not. The permutation test identified significant relations of the archaeal community composition with soil bacteria and actinobacteria quantities, diversity of micro-eukaryotes and archaea, soil pH and soil contents of C, N, P, Ca and Fe in conducive soil, while content of S was important in suppressive soil (Fig. 2C, Table S4).

The same pattern was obtained when considering discriminant archaeal OTUs (Metastats p < 0.05), as the two soils differed for 30 OTUs in bulk soil, 20 OTUs in cultivar Kariera and 27 OTUs in cultivar Agria, while within a same soil only a few OTUs separated one cultivar from the other, and from bulk soil (Fig. 3C).

The relative proportion of archaeal phyla did not differ between bulk soils and resistant cultivar Kariera. Yet, it differed between soils for susceptible cultivar Agria, which had about 60% of *Thaumarchaeota* and 40% of *Euryarchaeota* in conducive soil, versus 48% of *Thaumarchaeota* and 52% of *Euryarchaeota* in suppressive soil (Fig. 4B). Within these archaeal phyla, the same pattern was found for respectively the *Methanosarcinales* and *Nitrososphaerales* orders, and there was also an increase of *Nitrosotaleales* and *Methanomicrobiales* in suppressive soil (Fig. S3). Rarefaction curves showed that higher archaeal diversity occurred in conducive soil than in suppressive soil (two-way ANOVA; p < 0.001) and in the treatments the lowest diversity was in both cultivars in suppressive soil (two-way ANOVA; p < 0.05) (Fig. S2B, Table S7). When considering discriminant OTUs, differences were found mostly between the two soils, especially for *Thaumarchaeota* and *Euryarchaeota* OTUs. Suppressive soil was particularly enriched in 7 OTUs and conducive soil in 20 OTUs (Table S9). Significant correlative interactions (based on Spearman coefficient |ρ| ≥ 0.8) between archaeal OTUs were more numerous in tuberosphere of both cultivars than bulk soil but were also higher in suppressive soil than in conducive soil (Table S8).

In summary, Illumina sequencing of archaeal 16S rRNA genes showed major differences between conducive and suppressive soil, regardless of whether bulk soil, susceptible cultivar Agria or resistant cultivar Kariera were considered. The difference between cultivars was also significant but to a lesser extent. Four biotic and six abiotic factors increased with respect to archaea community in conducive soil, one increased in suppressive soil.

### Micro-eukaryotic community composition in bulk soil and tuberosphere by Illumina sequencing

A total of 1,244,356 18S rRNA gene sequences were obtained, out of which 896,483 (i.e. 72%) were mapped to 3,754 OTUs. The number of mapped sequences per sample ranged between 22291 and 51036 with a median at 42630. On a NMDS plot, samples from suppressive soil were relatively close to each other, whereas samples from conducive soil were more dispersed (Fig. 2D). In suppressive soil, there was relatively good separation of bulk soil, susceptible cultivar Agria and resistant cultivar Kariera, whereas treatments did not differ in conducive soil. The micro-eukaryotic communities differed overall (AMOVA, p = 0.006) and suppressive soil samples tended to differ from conducive soil samples, but this was significant only for bulk soils (AMOVA, p < 0.001). The permutation test identified significant relation between the micro-eukaryotic community and quantities of soil total bacteria and actinobacteria, diversity of archaea, soil pH and contents of C, N, P, Ca and Fe in conducive soil, and S content in suppressive soil. Above that, the diversity of micro-eukaryotes was higher in conducive bulk soil (Fig. 2D, Table S4).

Significantly different OTUs (Metastats p < 0.05) showed major differences between suppressive and conducive soils, with 258 discriminant OTUs for bulk soils, 327 for resistant cultivar Kariera, and 522 for susceptible cultivar Agria. Micro-eukaryotic communities differed least between bulk soil and cultivar Kariera in conducive soil (Fig. 3D).

There was a higher proportion of Ascomycota (Fig. 4C), in classes Pezizomycetes, Leotiomycetes, Eurotiomycetes, and particularly in Eurotiomycetes’ Chaetothyriales order (Fig. S4A) and Basidiomycota in suppressive bulk soil and a higher proportion of Chlorophyta, Ciliophora in classes Spirotrichea, Litostomatea and superclade CONThreeP (Fig. S4B), Myxogastria and Apicomplexa in conducive bulk soil. Compared with bulk soil, Chlorophyta and Cercozoa were in lower proportion with resistant cultivar (in conducive soils) and in similar proportion in both cultivars in suppressive soil. Basidiomycota were in higher proportion with both cultivars (in conducive soil), and the macro-eukaryotic phylum Arthropoda with cultivars Kariera (in conducive soil) and Agria (in suppressive soil) (Fig. 4C).

Rarefaction curves showed a slightly higher eukaryotic diversity in conducive soil than in suppressive soil overall, and diversity was generally lower in resistant cultivar than in susceptible cultivar, yet none of the differences was statistically significant (Fig. S2C; Table S7). Significant correlative interactions (based on Spearman coefficient |ρ| ≥ 0.8) between microeukaryotic OTUs were most numerous in tuberosphere of susceptible cultivar Agria but were at similar levels in resistant cultivar Kariera and bulk soil. They did not differ between the two soils (Table S8).

In summary, Illumina sequencing of eukaryotic 18S rRNA genes showed differences between conducive and suppressive soil but to some extent also between the cultivars, particularly for ciliates and fungi in conducive soil.

### Discriminant eukaryotic OTUs according to soil and potato cultivar

According to the differences observed at the level of eukaryotic phyla, OTUs from Ascomycota, Basidiomycota and Cercozoa were enriched in suppressive soil, while OTUs from Chlorophyta and Ciliophora were enriched in conducive soil. Specific OTUs of Ascomycota, Basidiomycota and Cercozoa were also prevalent in susceptible cultivar Agria in suppressive soil, while particularly OTUs of Chlorophyta and Ochrophyta were prevalent in cultivar Agria in conducive soil. Eukaryotic communities of resistant cultivar Kariera were separated in the two soils by OTUs of various phyla (Table S10).

## Discussion

Disease suppressiveness of Vyklantice soil L was shown in previous studies and was attributed to the interaction between potato plants and soil bacterial community^11,13^. In the current field experiment, previous work on common scab severity was extended by including two potato cultivars susceptible or resistant to CS. According to expectation, the CS severity of the susceptible cultivar grown in the suppressive soil was as low as for (i) the resistant cultivar in the same soil, and (ii) the resistant cultivar in the conducive soil. These results evidenced a similar potential of both types of CS control mechanisms. On one hand, suppressive soil was differentiated from conducive soil by (i) lower quantity of bacteria, and specifically of actinobacteria, (ii) a specific composition in archaea and microeukaryotes, and (iii) lower N, C, P, Ca, Fe contents and pH, and higher S content. Above that, in suppressive soil, higher Mg, P, Fe contents were found in the periderm of the resistant cultivar. On the other hand, the resistant cultivar Kariera differed from the susceptible cultivar Agria by (i) higher bacterial diversity, (ii) higher number of putative bacterial interactions, and (iii) specific bacterial community composition.

The quantity of pathogenic streptomycetes (based on numbers of *txtB* genes) did not change with soil suppressiveness status or cultivar in either tuberosphere or bulk soil, but in suppressive soil the number of pathogens decreased in potato periderm of both cultivars, possibly due to microbial interactions and soil chemical conditions depending on location^9,11^. It is consistent with previous observations that soil suppressivity is not related to pathogen quantity, which rather correlates with disease severity in compartments closest to the potato plant^9,11,14^ (Table S11).

Bacterial community structure has been often identified as a major factor in CS control^7,14^. In this work, microarray analysis evidenced mainly effects of suppressive vs conducive soil, with higher signals in suppressive soil for *Streptomyces* (*Actinobacteria*), *Bradyrhizobium*, *Burkholderia* (*Proteobacteria*), known to include plant-beneficial species and strains^3,26^ and *Nitrospira* (*Nitrospirae*), known for participation in nitrite oxidation^27^. In conducive soil, higher signals were detected for *Acidobacteria*, *Pseudomonas*, *Agrobacterium* and *Janithobacterium* (*Proteobacteria*), some of them also known for plant protection and antibiotic activities against fungi^28,29^. To some extent, Illumina sequencing discriminated between the two soils similarly to what microarray did, with a prevalence of *Bradyrhizobiaceae* (and other *Proteobacteria*), *Bacteroidetes* and *Firmicutes* in suppressive soil, and lower levels for different families of *Proteobacteria*, *Actinobacteria* and *Firmicutes* in conducive soil. However, Illumina sequencing showed that major effects were due to resistant vs susceptible cultivars. *Chloroflexi* and *Gaiellales* (*Actinobacteria*) were enriched in resistant cultivar Kariera, and *Burkholderia*, *Sphingomonas* (*Proteobacteria*) and *Actinobacteria* in susceptible cultivar Agria. Highest bacterial diversity was primarily associated with the resistant cultivar Kariera and it seems that high bacterial diversity may be of general importance in CS disease control because it was also observed in studies differing in methodological approaches^7^. Yet, various taxa were associated with low CS (i.e., suppressive soil or resistant cultivar) when comparing microbial communities from different field studies (Table S11) but increased proportion of actinobacteria and/or streptomycetes were observed repeatedly in healthy conditions. In this study, elevated numbers of actinobacteria occurred particularly in periderm of susceptible cultivar from conducive soil but those did not correspond to pathogenic streptomycetes. So perhaps, an antagonistic community of actinobacteria developed there as a response to pathogen infection, similarly as in Meng *et al*.^6^ or Tomihama *et al*.^30^.

Here, differences in bacterial communities were observed by two methodologies, as microarray pointed to general differences in soil community profiles, whereas Illumina sequencing enabled more detailed identification of bacterial OTUs, which highlighted lower taxonomic level differences between cultivars. This can be explained by the different focus of the two approaches. The microarray can assess only a limited number of community members, however, those are selected based on their specific ecological function and taxonomic hierarchy, so it directly tests functional hypotheses related to their presence/absence in the community. The Illumina sequencing can assess also unknown community members so it is more exhaustive, but represents only an observation of the microbial community^21–24,31,32^. Since the employed taxonomic microarray was designed to specifically target plant growth-promoting and antibiotic-producing bacterial taxa, it is of interest that those groups in particular differentiated between the suppressive and conducive soils^23,32^.

In the archaeal community, mainly the soil effects were evidenced. *Methanosarcinales* (*Euryarchaeota*), implicated in methylotrophic methanogenesis, were prevalent in both cultivars in suppressive soil and resistant cultivar Kariera in conducive soil, while *Nitrososphaerales*, (*Thaumarchaeota*), implicated in ammonia oxidation, were prevalent in both bulk soils and susceptible cultivar Agria in conducive soil. This might indicate changes in oxygen availability, which have been associated with *Nitrososphaerales* to *Methanosarcinales* ratio in situations of water level manipulation. Due to specific functions of the two archaeal groups, this may have further consequences for C and N cycling^33^.

The micro-eukaryotic community (especially fungi, parasitic Apicomplexa, Cercozoa, with weaker contributions from various bacterivores and autotrophs) differed between soils, as found with archaea and bacteria (especially with the microarray approach). Chlorophyta together with Myxogastria, Apicomplexa and Ciliophora were enriched in conducive soil, which consequently displayed increased micro-eukaryotic diversity and higher number of putative interactions. Enrichment of Chlorophyta suggests higher water content of that soil, which is compatible with a lower slope position^34^. Cercozoa and *Acanthamoeba* graze on bacteria^35^. Also, most Ciliophora are bacterivorous, but some species consume the content of fungal hyphae^36^. Certain Myxogastria species are fungivores, so they probably feed of the relatively abundant fungi in conducive soil and they might also affect bacterial-fungal dynamics^37^.

Feeding preferences may be reflected in the diversity and quantity of preys^15,17^ and in our study, differences were found in both, which raises the possibility of complex food-web interactions, potentially specific to soil and cultivar conditions, and suggests that top-down control of rhizosphere microbiome might be important to consider^36,38^. Trophic interactions between the domains can modify nutrient cycling and plant nutrition^15,35,36^, which can be relevant for soil suppressiveness^13,20^, with potential feedback effects of microbial communities themselves. In our study, content of P and Fe increased particularly by resistant cultivar accumulation so possibly, distinct microbial interactions occur also in various cultivars. As suppressive soil had lower N contents, this agrees also with the dominance of ammonia oxidizing archaea and suggests that N was recycled more intensively in suppressive than conducive soil^15^. This is also consistent with the possibility of enriched pathways of nitrogen metabolism in high CS conditions^14^ and high soil nitrogen content previously observed in CS conducive fields^11^.

Contents in Mg, S and P may influence the composition and functioning of microbial communities in potato rhizosphere^39,40^, and here we found differences in those nutrients between suppressive and conducive soils. A negative relationship between Mg periderm concentration and disease severity was found previously by Lazarovits *et al*.^41^, and similarly Lacey and Wilson^42^ found that CS disease severity was related to contents in exchangeable Ca, Mg, and K cations. Mg may by associated to phosphorus, which also agrees with increased P periderm concentration in healthy potatoes^10^.

Finally, several field studies have already compared microbiomes of soils and potato cultivars affected by common scab (Table S11). Although the approaches differed, it appearsthat low CS conditions were often associated with high bacterial diversity. Above that, bacterial interaction networks hypothesized from co-occurrence data were more complex, but those may be differently affected by predation and competition of organisms at higher trophic levels depending on soil conditions and climate. Suppressive bacterial communities were comprised of specific antinobacteria and/or streptomycetes, which is consistent with their antibiotic activities but also production of siderophores enabling better acquisition of iron and other metals^43,44^.

In conclusion of our study, microbiome features differed when comparing suppressive vs conducive soil as well as resistant vs susceptible cultivar^11,14^, but the relative importance of soil suppressiveness and cultivar resistance depended on the microbial community considered. Results suggest that the possible role of archaea and protists in suppressivity mechanisms deserves further attention. They also suggest that potential interactions between the three microbial domains under various field conditions would need to be considered for a comprehensive understanding of tuberosphere functioning and microbial CS control taking place in suppressive soils.

## Materials and Methods

### Sites

Vyklantice is a site where fields suppressive (49.5630N, 15.0575E; L for low disease severity) and conducive (49.5614N, 15.0546E; H for high disease severity) to potato CS occur at about 100 m distance. The two fields differ in common scab severity by observations over 30 years, while their geological context, soil type, climate and management are similar. The fields were regularly planted under a four-year crop rotation system including rapeseed, clover, potatoes, and grains (wheat or oats) in the past two decades^11^.

### Field experiment

Potatoes were planted in the beginning of May and sampled on July 16, 2009. Samples of bulk soil, tuberosphere soil and potatoes were collected. A CS susceptible cultivar Agria (Agrico Bohemia, Tabor, Czech Republic) and a resistant cultivar Kariera (Sativa Kerkov, Pribyslav, Czech Republic) were used. Potatoes were all certified seed tubers (common scab below 5% of surface). Four plots of each cultivar were planted at each field and the plots were arranged in a Latin square design. Each plot was planted with 3 rows of 12 potato plants (36 plants) separated by 50 cm of bare soil. Fields were fertilized with 100 kg N/ha (ammonium sulfate, 21% N), 35 kg P/ha (monocalcium phosphate, 35% P_2_O_5_), and 60 kg K/ha (potassium salt, 50% K_2_O). Potatoes were treated with pesticides, once with Nurelle D (EC) (chlorpyrifos, cypermethrin) 62 days after planting at 0.6 l/ha to prevent Colorado potato beetle (*Leptinotarsa decemlineata*), and twice with Acrobat MZ (dimethomorph, mancozeb), 48 and 62 days after planting at 2 kg/ha against the potato blight. Fungicides were not used.

### Sampling

One potato plant growing in the center of each plot was sampled, stored in a cooler and processed upon the arrival to the laboratory. Potato tubers from this plant were collected and washed in distilled water. All potatoes were carefully pealed using a sterile potato peeler (taking approximately 1 mm thick periderm samples), peels were homogenized, mixed and subsamples (1 subsample per plant) were stored in −80 °C and taken for further analyses (‘periderm’ samples). Tuberosphere soil samples of approximately 30 mL were collected no further than 3 mm from a potato tuber, filled to 50 mL falcon tubes, transferred to laboratory at −18 °C, and stored at −80 °C before the analysis (for details see^11^). Bulk soil of approximately 0.5 kg was collected at a distance of approximately 30 cm from the closest plant within each plot using a small sterile spade (1 sample per plot); part of it was used for chemical analysis and the rest was stored in Falcon tubes, as for rhizosphere soil. Common scab severity was evaluated on 20 potato tubers per plot using a 9-degree scale^45^. Potatoes used for evaluation were those of the collected plant and several more plants from each plot to achieve at least 20 measurements per plot.

### Soil and potato periderm analyses

The analytical methods were as previously described^11^. To determine total soil C, N, and S contents, 2-g samples of homogenized soil from both bulk soil and tuberosphere were dried, milled, and analyzed using Vario MAX CNS analyzer (Elementar Analysensysteme, Hanau, Germany). To determine all other elements, soil subsamples were leached with boiling nitro-hydrochloric acid (aqua regia) and assessed by optical emission spectroscopy with inductively coupled plasma (ICP-OES) by Aquatest Inc. (Prague, Czech Republic). Analyses of potato periderm were performed by service laboratory of the Institute of Botany (Trebon, Czech Republic). For total nitrogen analysis, 1–3 mg dried periderm was mineralized by modified Kjeldahl method in H_2_SO_4_ with catalyzer at 360 °C. For total phosphorus, 20 mg of dried periderm was sequentially decomposed by HNO_3_ and HClO_4_. In mineralized samples, both N and P were determined by flow injection analysis with spectrophotometric detection using FIA Lachat QC 8500 analyzer (Lachat Instruments, Hach Company, Loveland, CO). Cation contents in periderm were determined by atomic absorption spectrometry using AAS spectrometer ContrAA 700 (Analytik Jena, Jena, Germany) after mineralization with nitro-hydrochloric acid.

### Soil DNA extraction

Subsamples of 0.5 g tuberosphere and bulk soil were used for DNA extraction by method SK described by Sagova-Mareckova *et al*.^46^. Briefly, the method is based on bead-beating and phenol/chloroform extraction followed by purification with CaCl_2_ and GeneClean Turbo kit (MP Biomedicals, Santa Ana, CA). For DNA extraction from potato periderm, 3 g of periderm samples were fine cut in sterile Petri dish, homogenized, and a 0.3 g subsample was processed in the same way as soil samples to obtain total periderm DNA. DNA quantity and quality were evaluated using agarose gel and UV-absorption spectrometry with Nanophotometer (Implen, Munich, Germany).

### Real-time PCR (qPCR)

Quantifications were performed as previously described^11^ with primers eub338f/ eub518r^47,48^ amplifying a 197 bp fragment of the 16S rRNA gene from bacteria, act235f/eub518r^49^ yielding a 280 bp product for actinobacteria, and StrepF/StrepR yielding a 72 bp amplicon of the thaxtomin biosynthetic gene *txtB*^50^, respectively (Table 2). The analyses were done on a StepOne Plus Real-Time PCR System (Applied Biosystems, Foster City, CA) using 96-well plates with GoTaq qPCR Master Mix (Promega) containing SYBR Green as a double-stranded DNA binding dye. The reaction mixture contained in a total volume of 15 µl: 1× GoTaq qPCR Master Mix, 0.2 µM primers, and 0.2–2 ng diluted DNA sample. For all of the mentioned targets the PCR cycling protocol consisted of initial denaturation at 95 °C for 10 min, followed by 45 cycles of 95 °C for 15 s, 60 °C for 30 s and 72 °C for 30 s. Melting curves were recorded to ensure qPCR specificity. Baseline and threshold calculations were performed with the StepOne v. 2.2.2 software. The inhibition was tested by serial DNA dilution from each site, and the dilutions without inhibition of qPCR reactions were used for quantification. All qPCR measurements were done in duplicate. Standards for qPCR were prepared by cloning fragments of target genes from *Streptomyces europaeiscabiei* DSM 41802 in pGEM-T Easy vector system (Promega). After PCR verification and isolation of cloned constructs by Pure Yield Plasmid Miniprep System (Promega), a linear standard was prepared by cleaving with *Sal*I enzyme (New England Biolabs, UK) in a 200 µl reaction mixture containing 1× reaction buffer, 2 µg circular plasmid, and 20 U restriction endonuclease for 2 h in 37 °C. The linearized plasmid DNA was purified by phenol-chloroform extraction. Aliquots of linearized and purified standard diluted to 20 ng/µl were stored in −70 °C. Results were expressed per g dry soil. All results (including for *txtB*) were above detection limit.

**Table 2:** Primers used in qPCR and amplicon preparation for microarray and Illumina sequencing analyses.

Primer	Sequence (5′-3′)^a^	Sense	Target	Ref.
eub338f	ACTCCTACGGGAGGCAGCAG	forward	16S rRNA gene, bacteria	^47^
eub518r	ATTACCGCGGCTGCTGG	reverse		^48^
act235f	CGCGGCCTATCAGCTTGTTG	forward	16S rRNA gene, actinobacteria	^49^
StrepF	GCAGGACGCTCACCAGGTAGT	forward	*txtB* gene	^50^
StrepR	ACTTCGACACCGTTGTCCTCAA	reverse		
T7-pA	TAATACGACTCACTATAG-AGAGTTTGATCCTGGCTCAG	forward	16S rRNA gene, bacteria	^56^
pH	AAGGAGGTGATCCAGCCGCA	reverse		
CS1_515F	ACACTGACGACATGGTTCTACA-GTGCCAGCMGCCGCGGTAA	forward	16S rRNA gene, bacteria	^59^
CS2_806R	TACGGTAGCAGAGACTTGGTCT-GGACTACHVGGGTWTCTAAT	reverse		
CS1_ARC344F	ACACTGACGACATGGTTCTACA-AC-GGGGYGCAGCAGGCGCGA	forward	16S rRNA gene, archaea	^60^
CS2_Arch806R	TACGGTAGCAGAGACTTGGTCT-GG-ACTACVSGGGTATCTAAT	reverse		
CS1_Euk1391F	ACACTGACGACATGGTTCTACA -CG -GTACACACCGCCCGTC	forward	18S rRNA gene, eukaryotes	^61^
CS2_EukBr	TACGGTAGCAGAGACTTGGTCT-CA-TGATCCTTCTGCAGGTTCACCTAC	Reverse		

^a^The sequences aligning to the target are underlined for the primers with 5′ overhang parts.

### 16S rRNA gene-based taxonomic microarray

A taxonomic microarray based on DNA probes targeting 16S rRNA genes representing 19 bacterial phyla at different taxonomic levels^23^ was used to assess soil samples from potato fields. This microarray was validated previously^23,25^. Twelve probes targeting the genus *Streptomyces*, as well as *S. scabies* and relatives (Table 1) were added to the previous probe set (1033 probes) in this study. The probe KO 08^51^ for genus *Streptomyces* was obtained via the *probeBase* server^52^ (http://probebase.csb.univie.ac.at). The other 11 probes (20-mers) were designed in this study using ARB sofware^53^ (http://www.arb-home.de) and the parameters of the Probe Design function chosen by Sanguin *et al*.^31,54^. Probe specificity was tested with the Probe Match function in ARB against the reference Silva-104 and with the TestProbe online tool against Silva 126 database^55^ (http://www.arb-silva.de), at the weighted mismatch value of 1.5^23^. Hybridization properties of probes (e.g. melting temperature, potential formation of secondary structures and 3′dimers) were further tested *in silico*, according to Sanguin *et al*.^31,54^.

Universal bacterial primers T7-pA/pH (Table 2) were used to amplify 16S rRNA genes from total DNA extracts^56^. Primer T7-pA includes at the 5′ end the sequence of T7 promoter, which enabled T7 RNA polymerase-mediated *in vitro* transcription using purified PCR products as templates. PCR reactions were carried out using Taq Expand High Fidelity (Roche Applied Science, Meylan, France) and cycling conditions described in Kyselková *et al*.^23^. Purified PCR products (50 ng/µl) were fluorescently labelled (Cy3) by *in vitro* transcription, according to Stralis-Pavese *et al*.^57^. Purified RNA was fragmented by incubation with ZnSO_4_, as described^57^, and 400 ng subjected to hybridization on the microarray. Each probe was present in four copies per slide, and two slides were hybridized per sample.

Hybridization was carried out according to Sanguin *et al*.^31^. Slides were scanned at 532 nm, images were analyzed with GenePix Pro 7 (Molecular Devices, Sunnyvale, CA), and spot quality was checked visually, as described previously^31^. Data filtration was conducted using R 3.3.0^58^ (http://www.r-project.org). Hybridization of a given spot was considered positive when 80% of the spot pixels had intensity higher than the median local background pixel intensity plus twice the standard deviation of the local background. Intensity signals (median of signal minus background) were replaced by their square root value and intensity of each spot was then expressed as a fraction of the total intensity signal of the basic pattern it belongs to^54^. Finally, a given feature probe was considered as truly hybridized when (i) hybridization signals were superior to the mean signal of the negative controls and (ii) at least 3 of 4 replicate spots displayed positive hybridization^23^.

### Illumina MiSeq sequencing and analysis

From the DNA samples, fragments of the 16S rRNA gene including the variable region V4 were PCR amplified using universal primers with 5′linkers CS1_515F/CS2_806R^59^ for bacteria, and CS1_ARC344F/CS2_Arch806R^60^ for archaea (Table 2). PCRs were performed in 25 µl reaction volumes using the *Ex Taq* HS DNA Polymerase (Takara, Kusatsu, Japan), and the PCR conditions were as follows: 5 min initial denaturation at 95 °C, followed by 28 cycles of: 30 s denaturation at 95 °C, 45 s annealing at 55 °C for *Bacteria* or 50 °C for *Archaea*, and 30 s extension at 72 °C. Fragments of the eukaryotic18S rRNA gene including the variable region V9 were amplified using primers CS1_Euk1391F/CS2_EukBr^61^ (Table 2). PCR conditions were according to the standard protocol of the Earth microbiome project (http://www.earthmicrobiome.org): 3 min initial denaturation at 94 °C, followed by 28 cycles of: 45 s denaturation at 94 °C, 60 s annealing at 57 °C, and 90 s extension at 72 °C. Construction of amplicon libraries including the second PCR and sequencing using MiSeq sequencer (Illumina, San Diego, CA) were done at the DNA Services Facility, Research Resources Center, University of Illinois (Chicago, IL). Resulting paired sequence reads were merged, filtered, aligned using reference alignment from the Silva database^55^, and chimera checked using integrated Vsearch tool^62^ according to the MiSeq standard operation procedure (Miseq SOP, February 2018)^63^ in Mothur v. 1.39.5 software^64^. A taxonomical assignment of sequence libraries was performed in Mothur using the Silva Small Subunit rRNA Database, release 128^65^ adapted for use in Mothur (https://mothur.org/w/images/b/b4/Silva.nr_v128.tgz) as the reference database. Sequences of plastids, mitochondria, and those not classified in the domain Bacteria were discarded. The sequence library was clustered into OTUs using the Uparse pipeline in Usearch v10.0.240 software^21^, and the OTU table was further processed using tools implemented in the Mothur software. Distance matrices describing the differences in community composition between individual samples were calculated using the Yue-Clayton theta calculator^66^. Analysis of molecular variance (AMOVA)^67^ was based on a matrix of Yue-Clayton theta distances. Metastats analysis^68^ was used to detect differentially represented OTUs.

### Statistical analyses

Analysis of variance (ANOVA) and Fisher LSD tests were used to test differences between soils and cultivars for soil chemical parameters and log copy numbers of bacterial and actinobacterial 16S rRNA genes, and *txtB* genes in soil and periderm samples. Permutational multivariate analysis of variance (PERMANOVA) was used to compare distance matrices between microarray samples^69^. AMOVA was used to test differences between distance matrices (Yue-Clayton theta) between Illumina samples. The distance matrices were plotted by non-metric multidimensional scaling^70^ using Mass package, and vectors of environmental variables were fitted to the ordination using Vegan package in the R software environment^58^. Spearman’s correlation coefficients for OTUs were calculated using Hmisc package.

## Supplementary information

Supplementary file

## Data Availability

Illumina MiSeq amplicon sequences of 16S rRNA genes of bacteria and archaea, and 18S rRNA genes of eukaryotes are available in the NCBI Sequence Read Archive (www.ncbi.nlm.nih.gov/sra) as BioProject PRJNA474544. All other data is available in the Supplementary Material.
