# Supplementary material for: Bacterial, archaeal and micro-eukaryotic communities characterize a disease-suppressive or conducive soil and a cultivar resistant or susceptible to common scab
Source: Sci Rep. 2019 Oct 16;9:14883. doi: 10.1038/s41598-019-51570-6 (PMC6796001; doi:10.1038/s41598-019-51570-6)

Supplementary Information for

Bacterial, archaeal and micro-eukaryotic communities characterize a disease-suppressive or conducive soil and a cultivar resistant or susceptible to common scab.

Jan Kopecky, Zuzana Samkova, Ensyeh Sarikhani, Martina Kyselková, Marek Omelka, Vaclav Kristufek, Jiri Divis, Geneviève G. Grundmann, Yvan Moënne-Loccoz, Marketa Sagova-Mareckova

Corresponding author: Marketa Sagova-Mareckova,
Email: marketa.sagova@gmail.com

This PDF file includes:

Tables S1 to S11
Figs. S1 to S4

Supplementary Table S1. Quantitative real-time PCR analyses. Data were assessed by ANOVA and Fisher's LSD tests, and letters indicate significantly different samples ($p < 0.05$). L stands for suppressive and H for conducive Vyklantice soils.

A. Quantitative analyses of bacteria in bulk soil and tuberosphere samples. Data are shown as means with standard deviations (n = 4).

Sample	Cultivar / bulk soil	Bacterial 16S rRNA gene		Actinobacterial 16S rRNA gene		*txtB* gene	
		copies/g	SD	copies/g	SD	copies/g	SD
LB	bulk soil	9.69×10^{9} a,b	3.43×10^{9}	4.20×10^{9} a,b	1.02×10^{9}	8.64×10^{5}	1.12×10^{5}
LK	Kariera	1.38×10^{9} c	4.23×10^{8}	1.15×10^{9} c	4.65×10^{8}	6.85×10^{5}	5.80×10^{4}
LA	Agria	1.43×10^{9} b,c	2.37×10^{9}	2.27×10^{9} b	1.00×10^{9}	6.76×10^{5}	1.30×10^{4}
HB	bulk soil	1.37×10^{10} a	5.53×10^{9}	6.69×10^{9} a	2.50×10^{9}	6.34×10^{5}	2.09×10^{5}
HK	Kariera	1.14×10^{10} a,b	7.07×10^{9}	4.84×10^{9} a,b	3.03×10^{9}	7.17×10^{5}	1.33×10^{5}
HA	Agria	1.24×10^{10} a	6.19×10^{9}	4.85×10^{9} a,b	2.01×10^{9}	5.97×10^{5}	1.23×10^{5}

B. Quantitative analyses of bacteria in periderm samples. Data are shown as means with standard deviations (n = 4).

Sample	Cultivar	Actinobacterial 16S rRNA gene		*txtB* gene	
		copies/g	SD	copies/g	SD
LK	Kariera	3.31×10^{8} b	8.29×10^{7}	7.74×10^{5} b	4.84×10^{5}
LA	Agria	8.66×10^{7} b	5.44×10^{7}	1.21×10^{6} b	2.43×10^{5}
HK	Kariera	2.62×10^{8} a,b	1.53×10^{8}	4.61×10^{6} a,b	1.40×10^{6}
HA	Agria	1.61×10^{9} a	1.68×10^{9}	6.01×10^{7} a	6.72×10^{7}

Supplementary Table S2. Chemical analyses. Data were assessed by ANOVA and Fisher's LSD tests, and letters indicate significantly different samples ($p < 0.05$). L stands for suppressive and H for conducive Vyklantice soils.

A. Chemical analyses of bulk soil and tuberosphere samples. Data are shown as means ± standard deviations (n = 4).

**Sample**	**Cultivar / bulk soil**	**N [g/kg]**	**C [g/kg]**	**S [g/kg]**	**P [g/kg]**	**Mg [g/kg]**	**Ca [g/kg]**	**Fe [g/kg]**	**pH**
LB	bulk soil	1.67 ± 0.16 ^{b}	14.9 ± 2.0 ^{b}	0.642 ± 0.095 ^{b,c}	1.03 ± 0.08 ^{b,c}	11.2 ± 0.7	3.40 ± 0.78 ^{b}	39.5 ± 2.8 ^{b,c}	5.36 ± 0.16 ^{b}
LK	Kariera	1.67 ± 0.10 ^{b}	15.7 ± 0.9 ^{b}	0.855 ± 0.044 ^{a}	1.04 ± 0.06 ^{b,c}	12.0 ± 0.3	1.98 ± 0.10 ^{c}	38.6 ± 1.1 ^{c}	5.37 ± 0.15 ^{b}
LA	Agria	1.64 ± 0.12 ^{b}	15.4 ± 1.3 ^{b}	0.745 ± 0.091 ^{b}	1.00 ± 0.14 ^{c}	11.5 ± 0.5	1.81 ± 0.08 ^{c}	37.0 ± 2.0 ^{c}	5.36 ± 0.06 ^{b}
HB	bulk soil	2.24 ± 0.08 ^{a}	21.2 ± 1.1 ^{a}	0.661 ± 0.043 ^{b,c}	1.14 ± 0.06 ^{a,b}	11.3 ± 0.5	4.77 ± 0.32 ^{a}	39.7 ± 1.6 ^{b,c}	5.86 ± 0.16 ^{a}
HK	Kariera	2.29 ± 0.13 ^{a}	21.2 ± 1.3 ^{a}	0.623 ± 0.005 ^{c}	1.10 ± 0.04 ^{a,b,c}	10.7 ± 0.4	2.85 ± 0.36 ^{b}	42.6 ± 1.8 ^{a,b}	5.98 ± 0.08 ^{a}
HA	Agria	2.24 ± 0.08 ^{a}	20.9 ± 0.9 ^{a}	0.617 ± 0.014 ^{c}	1.21 ± 0.04 ^{a}	11.3 ± 0.5	2.94 ± 0.26 ^{b}	45.0 ± 1.9 ^{a}	5.99 ± 0.12 ^{a}

B. Chemical analyses of periderm samples. Data are shown as means ± standard deviations (n = 4).

**Sample**	**Cultivar**	**N [g/kg]**	**P [mg/kg]**	**Ca [g/kg]**	**Mg [g/kg]**	**Fe [g/kg]**
LK	Kariera	19.3 ± 1.8 ^{a,b}	2.85 ± 0.47	0.91 ± 0.20 ^{b}	0.793 ± 0.019 ^{a}	0.174 ± 0.049 ^{a}
LA	Agria	21.3 ± 2.0 ^{a}	3.00 ± 0.52	1.21 ± 0.08 ^{a}	0.652 ± 0.057 ^{b}	0.076 ± 0.005 ^{b}
HK	Kariera	14.7 ± 0.9 ^{c}	3.52 ± 0.47	1.04 ± 0.03 ^{a,b}	0.778 ± 0.063 ^{a}	0.091 ± 0.009 ^{b}
HA	Agria	16.0 ± 2.5 ^{b,c}	2.97 ± 0.31	1.24 ± 0.14 ^{a}	0.668 ± 0.046 ^{b}	0.076 ± 0.027 ^{b}

Supplementary Table S3. ANOVA of field (suppressive and conducive) and cultivar (resistant and susceptible) effects on data of soil and periderm chemical and microbial analyses. Levels of significance are indicated by asterisks, $p < 0.05$ *, $p < 0.01$ **, and $p < 0.001$ ***.

A. Soil analyses

	Df	N	C	S	P	Mg	Ca	Fe	pH	*Bacteria*	*Actinobacteria*	*txtB* gene
Field	1	0.021 ***	1.992 ***	77703 ***	101530 **	1475104	7537604 ***	98010417 ***	2.024 ***	6.78 ***	3.88 **	0.144
Cultivar	2	2.65E-05	0.003	15910	2117	58958	7647463 ***	4321667	0.012	2.78 *	1.74 *	0.037
Field × cultivar	2	1.68E-05	0.004	31708 **	10067	1903958	128904	30831667 **	0.011	1.20	0.38	0.076

B. Periderm analyses

	Df	N	P	Ca	Mg	Fe	*Actinobacteria*	*txtB* gene
Field	1	96.91 ***	412015	0.03	0.000184	0.0069 *	31.4 *	42.52 **
Cultivar	1	10.96	158614	0.23 *	0.05946 ***	0.0134 **	10.46	15.47
Field × cultivar	1	0.48	484017	0.01	0.000878	0.0063 *	0.08	0

Supplementary Table S4. Correlation between environmental variables and differences in community composition of bacteria, archaea, and micro-eukaryotes assessed by non-metric multidimensional scaling based on Bray-Curtis distance matrices. Levels of significance are indicated by dots, $p < 0.1$, and asterisks, $p < 0.05$ *, $p < 0.01$ **, and $p < 0.001$ ***.

Environmental variable		Microarray analysis			Illumina amplicon sequencing								
		bacteria			bacteria			archaea			micro-eukaryotes		
		r	p		r	p		r	p		r	p	
N	total content in tuberosphere soil	0.4501	0.006	**	0.1099	0.334		0.8073	0.001	***	0.5999	0.001	***
C		0.3950	0.013	*	0.1274	0.284		0.7728	0.001	***	0.5373	0.001	***
S		0.1533	0.202		0.0777	0.456		0.2259	0.096	.	0.2484	0.040	*
P		0.1001	0.343		0.0118	0.885		0.2818	0.059	.	0.1102	0.294	
Mg		0.2858	0.034	*	0.0109	0.902		0.1314	0.289		0.1495	0.205	
Ca		0.1794	0.139		0.2099	0.102		0.2636	0.071	.	0.2792	0.029	*
Fe		0.1432	0.208		0.0774	0.466		0.4105	0.010	**	0.3240	0.023	*
diversity of bacteria	area under the rarefaction curve	0.2173	0.090	.	0.7478	0.001	***	0.0019	0.973		0.0331	0.703	
diversity of micro-eukaryotes		0.4593	0.002	**	0.1174	0.295		0.5137	0.002	**	0.7220	0.001	***
diversity of archaea		0.2961	0.037	*	0.0121	0.887		0.5925	0.002	**	0.5459	0.001	***
pH		0.5751	0.002	**	0.2048	0.120		0.8889	0.001	***	0.6228	0.001	***
total bacteria	16S rRNA gene copies	0.0887	0.377		0.1490	0.236		0.5786	0.001	***	0.6104	0.001	***
actinobacteria		0.1399	0.208		0.1718	0.180		0.5610	0.002	**	0.5933	0.001	***
txtB	gene copies	0.2887	0.037	*	0.1960	0.128		0.1927	0.150		0.0979	0.307	

Supplementary Table S5. Average signal intensities of probes (n = 4)

A. Probes significantly separating the soils L and H (Metastats, $p < 0.05$)

Probe	Target group	LB	LK	LA	HB	HK	HA	p-value
Aceto3A	Some Acetobacteraceae	0.010	0.020	0.017	0.007	0.000	0.006	< 0.001
Acdp821	Acidiphilium	0.012	0.022	0.022	0.011	0.000	0.011	< 0.001
PalgiG3	Paenibacillus alginoliticus et rel. cluster	0.013	0.017	0.027	0.003	0.000	0.016	< 0.001
Pseu33	Pseudomonas citronellolis and Pseudomonas nitroreducens	0.016	0.033	0.021	0.022	0.000	0.011	0.001
Aci1	Acidiphillium	0.021	0.018	0.022	0.011	0.000	0.012	0.001
Strepto5	Streptomyces	0.009	0.020	0.012	0.012	0.000	0.014	0.003
Brady4	Bradyrhizobiaceae	0.030	0.034	0.038	0.034	0.000	0.030	0.005
StspSUB1	Streptosporangiales	0.003	0.011	0.015	0.002	0.000	0.013	0.007
Nit1B	Most Nitrosospira	0.000	0.016	0.022	0.008	0.000	0.013	0.013
Frank11	Frankia	0.000	0.005	0.007	0.000	0.000	0.003	0.037
Aceto3B	Acetobacteraceae	0.000	0.004	0.014	0.000	0.000	0.007	0.041
Burkho4B	Some Burkholderia	0.000	0.000	0.014	0.000	0.000	0.003	0.044
Glob2	Rhodopila globiformis	0.000	0.005	0.006	0.000	0.000	0.002	0.049

Probe	Target group	LB	LK	LA	HB	HK	HA	p-value
Janaga2	Janthinobacterium agaricidamnosum	0.000	0.000	0.012	0.024	0.045	0.016	0.005
Janaga3	Janthinobacterium agaricidamnosum	0.008	0.005	0.023	0.052	0.061	0.025	0.008
Acido-c	Uncultured Acidobacteria (Acidobacteria_4 cluster)	0.000	0.000	0.000	0.010	0.000	0.010	0.017
Barto2	Bartonella	0.000	0.000	0.000	0.003	0.006	0.009	0.020
RhizoLCSA2	Maize rhizosphere clones affiliated to Acidobacteria (Acidobacteria_7 cluster)	0.000	0.000	0.000	0.007	0.000	0.009	0.022
B6-603	Agrobacterium (G1, G3, G4, G7), A. rubi, A. larrymoorei, some Rhizobium and some Brevundimonas	0.000	0.000	0.000	0.000	0.000	0.008	0.026
MyxCor1	Myxococcus/Corallococcus	0.000	0.000	0.000	0.000	0.000	0.011	0.027
PseuD	Pseudomonas	0.000	0.000	0.000	0.015	0.000	0.014	0.034
AcidUnc	Uncultured Acidobacteria (Acidobacteria_6 cluster)	0.015	0.000	0.015	0.026	0.008	0.022	0.045

B. Probes significantly separating the two varieties A and K (Metastats, $p < 0.05$)

Probe	Target group	LB	LK	LA	HB	HK	HA	p-value
Strepto3	Streptomyces	0.022	0.037	0.020	0.021	0.039	0.014	< 0.001
Strepto1	Streptomyces	0.072	0.068	0.045	0.067	0.069	0.029	< 0.001
Strepto2	Streptomyces	0.059	0.052	0.034	0.046	0.055	0.020	< 0.001
Rzbc1247	Rhizobiaceae, Brucellaceae, Bartonella, Phyllobacteriaceae, Blastochloris, Azospirillum irakense and A. amazonense	0.037	0.044	0.033	0.059	0.061	0.027	0.001
BET940	Betaproteobacteria (except Comamonadaceae, Nitrosomonadaceae and Methylophilaceae)	0.060	0.051	0.046	0.054	0.043	0.037	0.001
Azo5	Azospirillum, some Roseomonas, Rhodospirillum, Rhodocista, Skermanella	0.009	0.017	0.017	0.021	0.023	0.016	0.003
Actino1	Streptosporangiales	0.017	0.025	0.012	0.013	0.016	0.007	0.003
Plancto4-mB	Most Planctomycetes	0.054	0.038	0.031	0.039	0.034	0.028	0.005
Rhizo157	Rhizobiaceae (except Agrobacterium), Bradyrhizobiaceae, Brucellaceae and Brevundimonas	0.068	0.064	0.033	0.058	0.024	0.025	0.011
Mycoba2	Mycobacterium	0.025	0.030	0.023	0.022	0.021	0.016	0.014
Mycoba1	Mycobacterium	0.025	0.028	0.021	0.021	0.024	0.018	0.019
CYA664	Most Cyanobacteria and some Chloroplasts	0.009	0.015	0.013	0.011	0.016	0.012	0.030
Kisp9	Kitasatospora griseola	0.012	0.025	0.023	0.028	0.022	0.020	0.032

Probe	Target group	LB	LK	LA	HB	HK	HA	p-value
Gludi	Gluconacetobacter diazotrophicus	0.000	0.000	0.021	0.000	0.000	0.015	< 0.001
TDRNO1030	Thermodesulforhabdus norvegica	0.000	0.000	0.012	0.003	0.000	0.013	< 0.001
Lacto39	Lactobacillus	0.000	0.000	0.006	0.000	0.000	0.007	< 0.001
Xan	Xanthobacter	0.000	0.000	0.018	0.000	0.000	0.021	< 0.001
Hyme3	Hymenobacter	0.000	0.000	0.008	0.000	0.000	0.011	< 0.001
Polycell	Polyangium cellulosum	0.000	0.000	0.009	0.000	0.000	0.011	< 0.001
Rhodobact1B	Rhodobacteraceae (excepted Paracoccus, Amaricoccus, Rhodobacter and Rhodovulum)	0.000	0.000	0.023	0.009	0.000	0.020	< 0.001
XAN818	Xanthomonas	0.000	0.000	0.020	0.000	0.000	0.009	< 0.001
Pho1	Photorhabdus	0.008	0.000	0.015	0.000	0.000	0.005	< 0.001
Acidocella1	Acidocella/Acidiphilium	0.006	0.004	0.020	0.011	0.000	0.022	0.001
Plancto12	Gemmata cluster	0.013	0.004	0.021	0.012	0.000	0.018	0.001
Stsp16	Streptosporangium	0.000	0.000	0.007	0.000	0.000	0.012	0.001
Ehrli1	Ehrlichiaceae (except Ehrlichia risticii and Cowdria)	0.000	0.000	0.006	0.000	0.000	0.012	0.001
Campy	Campylobacter	0.005	0.004	0.019	0.011	0.000	0.019	0.001
Bacpsf1	Bacillus pseudofirmus	0.000	0.000	0.006	0.000	0.000	0.010	0.001
Bkhcari1	Some Burkholderia (B. caribensis and B. hospita)	0.000	0.000	0.012	0.003	0.000	0.010	0.001
Dietz7	Dietzia	0.000	0.000	0.006	0.002	0.000	0.008	0.001
Nit1C	Some Nitrosospira	0.007	0.005	0.027	0.013	0.000	0.019	0.002
Polyang10	Polyangium	0.000	0.000	0.005	0.000	0.000	0.008	0.002
anaermycln	Acidobacteria (Subgroup 11)	0.000	0.000	0.007	0.004	0.000	0.010	0.002
pirelcln	Planctomycetaceae (Pir1 lineage)	0.000	0.000	0.014	0.009	0.000	0.014	0.003
Hypho5	Some Hyphomicrobium	0.000	0.004	0.011	0.000	0.000	0.009	0.005
Sphingo5B	Most Sphingomonadaceae	0.000	0.006	0.013	0.000	0.000	0.013	0.005
Delac3	Delftia acidovorans	0.000	0.000	0.004	0.000	0.000	0.009	0.006
Nso1225b	Betaproteobacteria ammonia oxidizers	0.000	0.000	0.006	0.000	0.000	0.008	0.006
Sacchps10	Saccharopolyspora	0.000	0.000	0.003	0.000	0.000	0.013	0.006
Cow1	Cowdria	0.000	0.000	0.004	0.000	0.000	0.014	0.007
Pagg6	Pantoea agglomerans	0.000	0.000	0.002	0.000	0.000	0.010	0.007
StreptomycesD7	Streptomyces	0.000	0.000	0.005	0.000	0.000	0.009	0.007
OP11-3	Uncultured eubacteria (OP11 division)	0.000	0.000	0.006	0.000	0.000	0.007	0.008
Bkand	Burkholderia andropogonis	0.000	0.000	0.006	0.000	0.000	0.012	0.008
Comtes2	Comamonas (mainly C. testosteroni)	0.000	0.000	0.005	0.000	0.000	0.010	0.008
Nancs7	Nannocystis	0.000	0.000	0.004	0.000	0.000	0.009	0.009
Rhi	Rhizobiaceae (except Agrobacterium)	0.000	0.000	0.002	0.000	0.000	0.014	0.011
Rhizo1B	Some Rhizobium	0.000	0.003	0.009	0.002	0.000	0.012	0.013
Spiro5	Spirosoma	0.000	0.000	0.002	0.000	0.000	0.005	0.024
Phyllobact	Mesorhizobium / Rhizobium	0.000	0.000	0.002	0.000	0.000	0.004	0.024
B6-603	Agrobacterium (G1, G3, G4, G7), A. rubi, A. larrymoorei, some Rhizobium and some Brevundimonas	0.000	0.000	0.000	0.000	0.000	0.008	0.025
Frtul7	Francisella tularensis	0.000	0.000	0.003	0.000	0.000	0.005	0.025
CystbSUB1	Cystobacterineae	0.000	0.000	0.003	0.004	0.000	0.008	0.026
MyxCor1	Myxococcus/Corallococcus	0.000	0.000	0.000	0.000	0.000	0.011	0.026
Plancto10	Some Pirrellula	0.000	0.000	0.002	0.000	0.000	0.008	0.028
Pagg5	Pantoea agglomerans	0.000	0.000	0.002	0.000	0.000	0.007	0.029
PseuD	Pseudomonas	0.000	0.000	0.000	0.015	0.000	0.014	0.033
Burkho4B	Some Burkholderia	0.000	0.000	0.014	0.000	0.000	0.003	0.034
PLA46	Planctomycetes, Lentipshaerae and OP3 candidate phylum	0.000	0.000	0.005	0.000	0.000	0.005	0.037
Nitmob	Nitrosococcus mobilis	0.000	0.000	0.004	0.000	0.000	0.005	0.038
Gloe2	Chlorogloeopsis	0.000	0.000	0.003	0.000	0.000	0.006	0.038
Aqutert	Aquicola tertiaricarbonis	0.000	0.000	0.003	0.004	0.000	0.008	0.039
Paen6	Paenibacillus	0.000	0.000	0.007	0.000	0.000	0.004	0.041
Diali14	Dialister	0.000	0.004	0.005	0.000	0.000	0.016	0.042
Baccir1	Bacillus circulans	0.000	0.000	0.008	0.000	0.000	0.008	0.043

Supplementary Table S6. Average frequencies (n = 4) of selected OTUs significantly contributing to separation of bacterial communities in the individual samples.

A. The most abundant OTUs significantly separating the soils L and H (Metastats, $p < 0.05$)

Group	LB	LK	LA	HB	HK	HA	p-value	Phylum	Class	Order	Family	Genus
Otu92	126	52	88	60	28	45	0,045	Proteobacteria	Betaproteobacteria	SC-I-84		
Otu253	115	61	104	68	27	55	0,015	Proteobacteria	Betaproteobacteria	SC-I-84		
Otu68	62	68	66	36	31	56	0,020	Proteobacteria	Alphaproteobacteria	Rhizobiales	Hyphomicrobiaceae	Devosia
Otu592	18	5	18	5	0	6	0,038	Proteobacteria	Alphaproteobacteria	Rhizobiales	Bradyrhizobiaceae	Rhodopseudomonas
Otu835	17	33	25	12	18	15	0,023	Proteobacteria	Deltaproteobacteria	Oligoflexales	0319-6G20	
Otu385	16	1	10	8	1	2	0,040	Planctomycetes	Phycisphaerae	Tepidisphaerales	Tepidisphaeraceae	
Otu3120	13	7	12	10	3	3	0,043	Proteobacteria	Alphaproteobacteria	Rhizobiales	Xanthobacteraceae	Labrys
Otu516	31	15	16	30	8	11	0,022	Proteobacteria	Gammaproteobacteria	Xanthomonadales	Xanthomonadaceae	Dokdonella
Otu1402	1	4	7	0	2	0	0,029	Bacteroidetes	Flavobacteriia	Flavobacteriales	Flavobacteriaceae	Chryseobacterium
Otu1154	0	5	9	2	2	0	0,012	Bacteroidetes	Flavobacteriia	Flavobacteriales	Flavobacteriaceae	Chryseobacterium
Otu2920	7	2	2	2	0	0	< 0.001	Proteobacteria	Deltaproteobacteria	Myxococcales	Polyangiaceae	Sorangium
Otu1408	6	7	6	7	4	1	0,047	Bacteroidetes	Sphingobacteriia	Sphingobacteriales	Chitinophagaceae	Flavisolibacter
Otu1343	4	1	4	2	1	0	0,031	FBP				
Otu2780	4	0	2	1	0	0	0,028	Planctomycetes	Phycisphaerae	Phycisphaerales	Phycisphaeraceae	
Otu3391	4	0	1	1	0	0	0,033	Firmicutes	Clostridia	Halanaerobiales	ODP1230B8.23	

Group	LB	LK	LA	HB	HK	HA	p-value	Phylum	Class	Order	Family	Genus
Otu355	16	17	22	21	33	32	0,040	Actinobacteria	MB-A2-108			
Otu369	32	18	28	22	39	37	0,008	Proteobacteria	Betaproteobacteria	Nitrosomonadales	Nitrosomonadaceae	
Otu1230	6	9	7	8	12	18	0,040	Actinobacteria	Thermoleophilia	Gaiellales		
Otu1478	5	6	5	9	11	9	0,045	Chloroflexi	TK10			
Otu886	5	6	3	7	11	7	0,033	Actinobacteria	Thermoleophilia	Solirubrobacterales		
Otu2120	7	8	10	4	14	17	0,018	Firmicutes	Bacilli	Bacillales	Planococcaceae	Sporosarcina
Otu899	7	3	4	20	1	1	0,019	Proteobacteria	Betaproteobacteria	SC-I-84		
Otu2391	2	1	1	5	6	1	0,015	Proteobacteria	Deltaproteobacteria	Myxococcales	Sandaracinaceae	
Otu2105	4	4	4	2	6	11	0,017	Firmicutes	Bacilli	Bacillales	Thermoactinomycetaceae	Shimazuella
Otu1621	2	3	2	3	6	5	0,012	unclas. Bacteria				
Otu1832	6	1	1	6	4	3	< 0.001	Proteobacteria	Alphaproteobacteria	Rhodospirillales	Rhodospirillaceae	Dongia
Otu2001	0	0	0	1	3	1	0,031	Proteobacteria	Deltaproteobacteria	Bdellovibrionales	Bdellovibrionaceae	Bdellovibrio
Otu1772	1	2	2	2	3	5	0,020	Firmicutes	Bacilli	Bacillales	Paenibacillaceae	Paenibacillus
Otu820	19	7	8	12	13	13	0,002	Proteobacteria	Deltaproteobacteria	Myxococcales	Haliangiaceae	Haliangium
Otu3265	1	2	0	2	3	2	0,045	Cyanobacteria	ML635J-21			

B. The most abundant OTUs significantly separating the varieties Kariera (K) and Agria (A) (Metastats, $p < 0.05$)

Group	LB	LK	LA	HB	HK	HA	p-value	Phylum	Class	Order	Family	Genus
Otu4	347	956	374	576	1177	386	< 0.001	Chloroflexi	KD4-96			
Otu16	13	435	7	198	615	24	< 0.001	Actinobacteria	Thermoleophilia	Gaiellales	Gaiellaceae	Gaiella
Otu55	406	766	464	504	1011	450	0,019	Chloroflexi	KD4-96			
Otu48	24	283	9	155	278	15	< 0.001	Proteobacteria	Alphaproteobacteria	Sphingomonadales	Sphingomonadaceae	Sphingomonas
Otu12	96	360	101	233	439	165	0,001	Actinobacteria	Thermoleophilia	Gaiellales	Gaiellaceae	Gaiella
Otu51	77	303	49	269	273	42	< 0.001	Acidobacteria	Subgroup_6			
Otu164	59	249	55	121	328	61	< 0.001	Chloroflexi	KD4-96			
Otu30	6	215	2	127	243	15	< 0.001	Actinobacteria	Thermoleophilia	Gaiellales	Gaiellaceae	Gaiella
Otu69	22	195	21	98	238	37	< 0.001	Actinobacteria	Thermoleophilia	Solirubrobacterales	288-2	
Otu46	2	162	3	105	183	5	< 0.001	Actinobacteria	Thermoleophilia	Gaiellales		
Otu11	281	252	93	450	221	49	0,039	Proteobacteria	Alphaproteobacteria	Rhizobiales	Xanthobacteraceae	
Otu29	17	164	8	75	169	10	< 0.001	Chloroflexi	Gitt-GS-136			
Otu275	149	252	113	290	242	99	0,002	Acidobacteria	Subgroup_6			
Otu107	1	106	0	53	166	1	< 0.001	Actinobacteria	Thermoleophilia	Gaiellales	Gaiellaceae	Gaiella
Otu38	153	213	62	295	152	34	0,005	Proteobacteria	Betaproteobacteria	Burkholderiales	Comamonadaceae	Piscinibacter
Otu33	41	165	12	129	131	18	< 0.001	Proteobacteria	Gammaproteobacteria	Xanthomonadales	Xanthomonadaceae	Arenimonas
Otu105	2	125	2	58	140	5	< 0.001	Actinobacteria	Thermoleophilia	Gaiellales		
Otu696	6	164	8	94	110	10	< 0.001	Proteobacteria	Alphaproteobacteria	Sphingomonadales	Sphingomonadaceae	Sphingomonas
Otu143	42	145	20	162	145	19	< 0.001	Acidobacteria	Subgroup_6			
Otu141	0	110	0	42	142	1	< 0.001	Actinobacteria	MB-A2-108			
Otu123	17	123	11	75	150	17	< 0.001	Chloroflexi	Chloroflexia	Chloroflexales	Roseiflexaceae	Roseiflexus
Otu24	70	167	77	118	234	81	< 0.001	Actinobacteria	Thermoleophilia	Gaiellales	Gaiellaceae	Gaiella
Otu64	239	193	67	413	141	31	0,026	Verrucomicrobia	Spartobacteria	Chthoniobacterales	DA101_soil_group	
Otu61	11	101	8	68	142	10	< 0.001	Proteobacteria	Betaproteobacteria	Nitrosomonadales	Nitrosomonadaceae	
Otu283	60	141	53	102	196	60	0,002	Chloroflexi	KD4-96			
Otu74	2	105	6	42	132	8	< 0.001	Chloroflexi	Thermomicrobia	JG30-KF-CM45		
Otu282	10	122	9	66	117	10	< 0.001	Proteobacteria	Alphaproteobacteria	Sphingomonadales	WW2-159	
Otu73	63	151	36	202	119	32	0,005	Acidobacteria	Subgroup_6			
Otu138	21	131	29	62	127	37	< 0.001	Actinobacteria	Actinobacteria	Propionibacteriales	Nocardioidaceae	Nocardioides
Otu76	80	140	29	206	96	19	0,006	Acidobacteria	Blastocatellia	Blastocatellales	Blastocatellaceae_Subgr.4	RB41

Group	LB	LK	LA	HB	HK	HA	p-value	Phylum	Class	Order	Family	Genus
Otu1	1213	687	1980	746	522	1971	< 0.001	Proteobacteria	Alphaproteobacteria	Sphingomonadales	Sphingomonadaceae	Sphingomonas
Otu21	633	145	841	236	169	836	< 0.001	Actinobacteria	Thermoleophilia	Gaiellales		
Otu6	288	67	558	86	73	652	< 0.001	Actinobacteria	Actinobacteria	Propionibacteriales	Nocardioidaceae	Nocardioides
Otu140	178	13	306	24	23	341	< 0.001	Actinobacteria	Thermoleophilia	Gaiellales		
Otu18	397	50	323	127	46	370	< 0.001	Chloroflexi	JG37-AG-4			
Otu63	176	56	311	56	62	370	< 0.001	Actinobacteria	Actinobacteria	Frankiales	Frankiaceae	Jatrophihabitans
Otu41	160	33	263	44	39	342	< 0.001	Actinobacteria	Thermoleophilia	Gaiellales		
Otu114	221	38	315	85	46	298	< 0.001	Actinobacteria	Thermoleophilia	Gaiellales		
Otu104	212	35	303	60	30	263	< 0.001	Actinobacteria	Thermoleophilia	Gaiellales		
Otu19	183	8	257	25	12	247	< 0.001	Actinobacteria	Thermoleophilia	Gaiellales		
Otu20	147	35	241	69	47	314	< 0.001	Actinobacteria	Actinobacteria	Frankiales	Acidothermaceae	Acidothermus
Otu31	160	16	246	37	26	263	< 0.001	Actinobacteria	Thermoleophilia	Solirubrobacterales	TM146	
Otu54	175	45	248	67	55	267	< 0.001	Actinobacteria	Actinobacteria	Frankiales	uncultured	
Otu84	73	3	191	5	1	228	0,001	Proteobacteria	Gammaproteobacteria	Xanthomonadales	Xanthomonadaceae	Rhodanobacter
Otu26	138	8	215	15	11	198	< 0.001	Chloroflexi	Ktedonobacteria	Ktedonobacterales	Ktedonobacteraceae	
Otu8	221	195	387	162	253	439	0,016	Actinobacteria	Thermoleophilia	Gaiellales		
Otu309	109	6	194	14	5	186	< 0.001	Actinobacteria	Thermoleophilia	Gaiellales		
Otu13	139	93	248	106	97	275	< 0.001	Actinobacteria	Actinobacteria	Micrococcales	Intrasporangiaceae	Oryzihumus
Otu10	181	136	308	127	106	255	< 0.001	Actinobacteria	Actinobacteria	Micrococcales	Intrasporangiaceae	Terrabacter
Otu137	82	13	166	12	12	180	< 0.001	Chloroflexi	Ktedonobacteria	C0119		
Otu50	89	14	160	22	20	187	< 0.001	Proteobacteria	Alphaproteobacteria	Rhodospirillales	Rhodospirillales Inc. Sedis	Cand. Alysiosphaera
Otu176	63	34	187	44	38	175	< 0.001	Actinobacteria	Actinobacteria	Streptomycetales	Streptomycetaceae	Streptomyces
Otu199	93	9	160	14	5	140	< 0.001	Proteobacteria	Betaproteobacteria	Burkholderiales	Burkholderiaceae	(Para)Burkholderia
Otu36	350	15	197	112	4	100	< 0.001	Gemmatimonadetes	Gemmatimonadetes	Gemmatimonadales	Gemmatimonadaceae	
Otu52	213	22	186	73	22	128	< 0.001	Actinobacteria	Thermoleophilia	Gaiellales	Gaiellales_unclassified	
Otu49	110	17	163	31	21	144	< 0.001	Actinobacteria	Thermoleophilia	Gaiellales		
Otu77	59	18	135	23	18	160	< 0.001	Chloroflexi	Ktedonobacteria	C0119		
Otu23	204	143	271	196	188	302	0,014	Actinobacteria	Thermoleophilia	Gaiellales	Gaiellaceae	Gaiella
Otu946	136	52	192	76	69	171	< 0.001	Actinobacteria	Thermoleophilia	Gaiellales		
Otu117	44	8	108	17	11	139	< 0.001	Actinobacteria	Actinobacteria	Frankiales	Geodermatophilaceae	Modestobacter

Supplementary Table S7. Effects of site, treatment, and their interaction on diversity of microbial communities. Two-way analysis of variance, n=4. Levels of significance are indicated by dots, $p < 0.1$, or asterisks, $p < 0.05$ *, $p < 0.01$ **, and $p < 0.001$ ***.

Bacteria

	Sum Sq	Df	F value	Pr(>F)
site	39544	1	2.6604	0.1224
treatment	496817	2	16.7125	0.0001 ***
site:treatment	95263	2	3.2046	0.0675 .
Residuals	237818	16		

Microeukaryotes

	Sum Sq	Df	F value	Pr(>F)
site	77255	1	1.8266	0.1933
treatment	253156	2	2.9928	0.0755 .
site:treatment	72860	2	0.8613	0.4393
Residuals	761301	18		

Archaea

	Sum Sq	Df	F value	Pr(>F)
site	2660.84	1	47.6212	5.1e-06 ***
treatment	445.64	2	3.9878	0.0409 *
site:treatment	194.94	2	1.7176	0.2130
Residuals	838.13	15		

Supplementary Table S8. Correlations between proportions of individual OTUs in the communities of *Bacteria, Archaea,* and microeukaryotes (Spearman, |ρ| ≥ 0.8).

Bacteria	**Rhizosphere**		**Bulk soil**	**L soil**	**H soil**
	**Agria**	**Kariera**			
OTUs (min in two samples)	2942	3335	3416	3682	3778
Number of correlations	181026	332847	588300	415347	508320
Avg number of neighbors	61.5	99.8	172.2	112.8	134.5
Positive correlations	110401	174899	352691	254492	285632
Negative correlations	70625	157948	235609	160855	222688

Archaea	**Rhizosphere**		**Bulk soil**	**L soil**	**H soil**
	**Agria**	**Kariera**			
OTUs (min in two samples)	87	80	88	95	69
Number of correlations	579	672	370	200	133
Avg number of neighbors	6.7	8.4	4.2	2.1	1.9
Positive correlations	426	453	256	142	116
Negative correlations	153	219	114	58	17

**Microeukaryotes**	**Rhizosphere**		**Bulk soil**	**L soil**	**H soil**
	**Agria**	**Kariera**			
OTUs (min in two samples)	2339	1801	2148	2495	2229
Number of correlations	142260	67000	72585	20828	16411
Avg number of neighbors	60.8	37.2	33.8	8.3	7.4
Positive correlations	107366	55606	62538	19561	15286
Negative correlations	34894	11394	10047	1267	1125

Supplementary Table S9. Average frequencies (n = 4) of selected OTUs significantly contributing to separation of archaeal communities in the individual samples.

A. The most abundant OTUs significatly separating the soils L and H (Metastats, $p < 0.05$)

Group	LB	LK	LA	HB	HK	HA	p-value	Phylum	Class	Order	Family	Genus
Otu1	6626	11846	11496	5229	6200	5634	0.024	Euryarchaeota	Methanomicrobia	Methanosarcinales	Methanosarcinaceae	Methanosarcina
Otu3	6028	3736	4132	1784	2311	701	0.009	Thaumarchaeota	Nitrososphaeria	Nitrososphaerales	Nitrososphaeraceae	
Otu5	869	1033	986	428	292	498	0.001	Euryarchaeota	Methanomicrobia	Methanomicrobiales	Methanomicrobiaceae	Methanoculleus
Otu6	1153	596	707	359	462	238	0.028	Thaumarchaeota	Nitrososphaeria	Nitrososphaerales	Nitrososphaeraceae	
Otu10	584	311	223	1	12	1	0.001	Thaumarchaeota	Nitrososphaeria	Nitrosotaleales	Nitrosotaleaceae	Cand. Nitrosotalea
Otu17	429	426	503	90	105	36	0.001	Thaumarchaeota	Nitrososphaeria	Nitrososphaerales	Nitrososphaeraceae	
Otu93	97	102	130	53	38	41	0.001	Euryarchaeota	Methanomicrobia	Methanosarcinales	Methanosarcinaceae	Methanosarcina
Otu29	11	7	30	1	2	0	0.003	Thaumarchaeota	Group_c			
Otu42	8	3	13	0	0	0	0.001	Euryarchaeota	Thermoplasmata	Methanomassiliicoccales	uncultured	
Otu69	1	3	2	0	1	0	0.032	Thaumarchaeota	Nitrososphaeria	Nitrososphaerales	Nitrososphaeraceae	Cand. Nitrocosmicus

Group	LB	LK	LA	HB	HK	HA	p-value	Phylum	Class	Order	Family	Genus
Otu38	1	0	6	31	9	32	0.006	Thaumarchaeota	Nitrososphaeria	Nitrososphaerales	Nitrososphaeraceae	
Otu91	6	1	1	23	15	36	0.001	Thaumarchaeota	Nitrososphaeria	Nitrososphaerales	Nitrososphaeraceae	
Otu36	0	0	0	17	13	41	0.001	Thaumarchaeota	Nitrososphaeria	Nitrososphaerales	Nitrososphaeraceae	
Otu55	17	5	4	46	38	36	0.015	Thaumarchaeota	Nitrososphaeria	Nitrososphaerales	Nitrososphaeraceae	
Otu96	0	0	0	20	46	23	0.002	Euryarchaeota	Thermoplasmata	uncultured		
Otu22	7	0	0	40	47	20	0.002	Nanoarchaeaeota	Woesearchaeia			
Otu76	0	0	0	18	64	14	0.001	Euryarchaeota	Thermoplasmata	unclass.		
Otu25	0	0	0	27	38	36	0.001	Euryarchaeota	Thermoplasmata	uncultured		
Otu23	0	0	0	23	40	47	0.001	Thaumarchaeota	Nitrososphaeria	Nitrososphaerales	Nitrososphaeraceae	Cand. Nitrososphaera
Otu87	0	0	0	19	79	19	0.001	Euryarchaeota	Thermoplasmata	uncultured		
Otu20	2	0	0	33	53	40	0.001	Euryarchaeota	Thermoplasmata	uncultured		
Otu31	0	0	0	35	71	18	0.002	Euryarchaeota	Thermoplasmata	uncultured		
Otu86	68	18	11	83	62	135	0.016	Thaumarchaeota	Nitrososphaeria	Nitrososphaerales	Nitrososphaeraceae	
Otu65	0	0	0	42	100	47	0.001	Euryarchaeota	Thermoplasmata	uncultured		
Otu40	2	0	1	59	124	52	0.001	Euryarchaeota	Thermoplasmata	unclass.		
Otu39	1	0	0	54	115	75	0.001	Euryarchaeota	Thermoplasmata	uncultured		
Otu14	0	0	0	48	143	53	0.001	Euryarchaeota	Thermoplasmata	uncultured		
Otu78	12	0	12	91	134	85	0.001	Euryarchaeota	Thermoplasmata	uncultured		
Otu19	0	0	1	63	168	59	0.001	Euryarchaeota	Thermoplasmata	uncultured		
Otu61	4	0	2	115	87	187	0.002	Thaumarchaeota	Nitrososphaeria	Nitrososphaerales	Nitrososphaeraceae	
Otu15	71	10	50	164	246	126	0.013	Euryarchaeota	Thermoplasmata	uncultured		
Otu18	5	1	3	112	102	216	0.001	Thaumarchaeota	Nitrososphaeria	Nitrososphaerales	Nitrososphaeraceae	
Otu13	4	0	0	249	164	317	0.001	Thaumarchaeota	Nitrososphaeria	Nitrososphaerales	Nitrososphaeraceae	Cand. Nitrososphaera
Otu70	18	6	2	303	217	480	0.002	Thaumarchaeota	Nitrososphaeria	Nitrososphaerales	Nitrososphaeraceae	
Otu12	30	1	9	498	1166	493	0.001	Euryarchaeota	Thermoplasmata	uncultured		

Otu64	372	117	250	1149	1289	829	0.006	Euryarchaeota	Methanomicrobia	Methanosarcinales	Methanosarcinaceae	Methanosarcina
Otu4	765	296	285	1378	1495	1803	0.002	Thaumarchaeota	Nitrososphaeria	Nitrososphaerales	Nitrososphaeraceae	
Otu9	5	0	4	1655	779	1598	0.001	Thaumarchaeota	Nitrososphaeria	Nitrososphaerales	Nitrososphaeraceae	
Otu8	201	47	30	1250	1965	1146	0.001	Euryarchaeota	Thermoplasmata	unclass.		
Otu7	106	22	37	1722	1957	2537	0.001	Thaumarchaeota	Nitrososphaeria	Nitrososphaerales	Nitrososphaeraceae	

B. The most abundant OTUs significatly separating the varieties Kariera (K) and Agria (A) (Metastats, $p < 0.05$)

Overall comparison

Group	LB	LK	LA	HB	HK	HA	p-value	Phylum	Class	Order	Family	Genus
Otu112	0	0	0	4	1	0	0.045	Nanoarchaeaeota	Woesearchaeia	unclass.		
Otu75	0	0	0	0	1	0	0.045	Euryarchaeota	Methanobacteria	Methanobacteriales	Methanobacteriaceae	Methanobrevibacter
Otu92	0	0	0	0	0	2	0.034	Euryarchaeota	Thermoplasmata	uncultured		
Otu73	55	13	49	34	9	23	0.009	Euryarchaeota	Methanomicrobia	Methanomicrobiales	Methanomicrobiaceae	Methanoculleus

Comparison of the varieties in conducive soil H

Group	LB	LK	LA	HB	HK	HA	p-value	Phylum	Class	Order	Family	Genus
Otu64	372	117	250	879	1696	829	0.019	Euryarchaeota	Methanomicrobia	Methanosarcinales	Methanosarcinaceae	Methanosarcina
Otu17	429	426	503	170	4	36	0.012	Thaumarchaeota	Nitrososphaeria	Nitrososphaerales	Nitrososphaeraceae	unclass.
Otu43	74	40	25	159	7	40	0.039	Thaumarchaeota	Nitrososphaeria	Nitrososphaerales	Nitrososphaeraceae	unclass.

Comparison of the varieties in suppressive soil L

Group	LB	LK	LA	HB	HK	HA	p-value	Phylum	Class	Order	Family	Genus
Otu29	11	7	30	2	0	0	0.05	Thaumarchaeota	Group_c			
Otu73	55	13	49	34	9	23	0.018	Euryarchaeota	Methanomicrobia	Methanomicrobiales	Methanomicrobiaceae	Methanoculleus

Supplementary Table S10. Average frequencies (n = 4) of selected OTUs significantly contributing to separation of eukaryotic communities in the individual samples.

A. The most abundant OTUs significatly separating the soils L and H (Metastats, $p < 0.05$)

Group	LB	LK	LA	HB	HK	HA	p-value	Phylum	Class	Order	Family	Genus
Otu1	1044	2101	1952	400	500	603	0.001	Ascomycota	Eurotiomycetes	Chaetothyriales	unclass.	
Otu10	581	855	688	358	255	564	0.02	Ascomycota	Leotiomycetes	Incertae_Sedis	Pseudeurotiaceae	Pseudogymnoascus
Otu25	452	488	586	227	134	267	0.001	Schizoplasmodiida	Schizoplasmodiida_cl	Schizoplasmodiida_or	Schizoplasmodiida_fa	Ceratiomyxella
Otu34	306	144	177	57	15	40	0.001	Basidiomycota	Tremellomycetes	Tremellales	Trimorphomycetaceae	Saitozyma
Otu36	119	234	232	32	9	37	0.001	Cercozoa	unclass.			
Otu47	54	405	65	2	15	5	0.001	Basidiomycota	Tremellomycetes	Tremellales	Rhynchogastremataceae	Papiliotrema
Otu45	60	210	223	0	0	2	0.001	unclass.				
Otu80	91	151	198	9	6	6	0.001	Ascomycota	Eurotiomycetes	Chaetothyriales	Trichomeriaceae	Knufia
Otu74	84	257	113	7	16	12	0.001	Ascomycota	Pezizomycetes	Pezizales	Pyronemataceae	Melastiza
Otu182	119	220	230	56	30	95	0.001	unclass.				
Otu51	251	73	61	1	0	0	0.002	Nematoda	Chromadorea	unclass.		
Otu59	221	92	66	3	5	12	0.001	Mucoromycota	Incertae_Sedis	Mucorales	Mucoraceae	Mucor
Otu1080	95	169	118	8	12	12	0.001	Ascomycota	Leotiomycetes	unclass.		
Otu69	1	152	169	0	2	1	0.007	Arthropoda	Arachnida	Acari_or	Acari_fa	Acari_ge
Otu68	31	121	168	1	6	1	0.001	Ascomycota	Pezizomycetes	Pezizales	Pyronemataceae	unclass.
Otu42	128	160	128	46	28	44	0.001	Ochrophyta	unclass.			
Otu41	77	230	150	57	63	48	0.008	unclass.				
Otu117	167	45	45	8	8	2	0.001	Klebsormidiophyceae	Klebsormidiophyceae_cl	Klebsormidiales	Klebsormidiales_fa	Klebsormidium
Otu97	136	172	164	56	80	99	0.006	Discosea	Longamoebia	Centramoebida	Centramoebida_fa	Acanthamoeba
Otu76	28	102	121	5	7	5	0.001	Basidiomycota	Agaricomycetes	Agaricales	uncultured	uncultured_ge
Otu102	97	130	5	0	0	0	0.001	Basidiomycota	Agaricomycetes	Trechisporales	Hydnodontaceae	Trechispora
Otu29	147	247	213	131	65	183	0.044	Ochrophyta	Xanthophyceae	Tribonematales	Tribonematales_fa	Xanthonema
Otu108	136	102	52	10	42	20	0.009	unclass.				
Otu1590	67	81	84	4	8	6	0.001	Ascomycota	Leotiomycetes	Helotiales	uncultured	
Otu2050	39	107	44	5	2	1	0.001	Mucoromycota	Glomeromycetes	Paraglomerales	Paraglomeraceae	Paraglomus
Otu49	73	129	117	82	21	44	0.004	Cercozoa	Glissomonadida	Glissomonadida_or	Glissomonadida_fa	Glissomonadida_ge
Otu115	44	41	86	0	0	0	0.001	Cercozoa	Phytomyxea	Phytomyxea_or	Phytomyxea_fa	Plasmodiophora
Otu103	36	90	71	4	6	20	0.002	Schizoplasmodiida	Schizoplasmodiida_cl	Schizoplasmodiida_or	Schizoplasmodiida_fa	Ceratiomyxella
Otu148	46	60	54	6	3	7	0.001	unclass.				
Otu96	106	56	91	37	28	47	0.01	Cercozoa	unclass.			

Group	LB	LK	LA	HB	HK	HA	p-value	Phylum	Class	Order	Family	Genus
Otu105	28	5	9	47	41	57	0.002	unclass.				
Otu1934	5	0	1	16	82	17	0.004	Ciliophora	Intramacronucleata	Litostomatea	Haptoria	Arcuospathidium
Otu223	1	1	5	4	56	57	0.005	unclass.				
Otu159	27	5	3	36	34	81	0.005	unclass.				
Otu146	0	0	0	59	43	18	0.001	unclass.				
Otu160	14	4	5	20	108	18	0.046	Ochrophyta	Diatomea	unclass.		
Otu155	12	5	16	42	76	43	0.001	Peronosporomycetes	Peronosporomycetes_cl	Peronosporomycetes_or	Peronosporomycetes_fa	Aplanopsis
Otu124	15	17	19	16	69	96	0.006	Ochrophyta	Chrysophyceae	Chromulinales	Chromulinales_fa	Spumella
Otu1245	15	7	12	79	21	67	0.007	Cercozoa	Thecofilosea	Cryomonadida	Rhizaspididae	Rhogostoma
Otu268	0	0	0	31	55	49	0.001	unclass.				
Otu120	1	6	3	66	27	58	0.003	Mucoromycota	Incertae_Sedis	Mucorales	Rhizopodaceae	Rhizopus
Otu192	3	0	3	27	58	65	0.001	Cercozoa	Cercomonadidae	Cercomonadidae_or	Cercomonadidae_fa	Cercomonas
Otu119	22	10	19	102	35	70	0.002	unclass.				
Otu86	31	14	24	102	43	82	0.001	Ciliophora	Intramacronucleata	Conthreep	unclass.	
Otu81	20	11	11	91	35	80	0.001	Chlorophyta_ph	Chlorophyceae	Chlamydomonadales	Chlamydomonadales_fa	Fasciculochloris
Otu87	65	18	38	161	51	77	0.016	Ochrophyta	Eustigmatophyceae	Eustigmatales	Eustigmatales_fa	Eustigmatos
Otu75	25	8	17	126	28	69	0.003	unclass.				
Otu77	27	14	10	117	81	53	0.004	Ciliophora	Intramacronucleata	Litostomatea	Haptoria	unclass.
Otu46	43	24	52	121	72	149	0.015	Myxogastria	Myxogastria_cl	Myxogastria_or	Myxogastria_fa	Lepidoderma
Otu116	0	0	0	0	231	0	0.001	Basidiomycota	Agaricomycetes	Agaricales	Marasmiaceae	Marasmius
Otu84	47	24	21	189	142	65	0.002	Ciliophora	Intramacronucleata	Conthreep	unclass.	
Otu31	102	40	40	275	57	158	0.011	Chlorophyta_ph	Chlorophyceae	Chlamydomonadales	Chlamydomonadales_fa	Tetracystis
Otu40	66	8	31	122	87	240	0.001	Cercozoa	Phytomyxea	Phytomyxea_or	Phytomyxea_fa	Plasmodiophora
Otu57	3	0	2	100	90	174	0.001	unclass.				
Otu89	28	28	57	319	78	125	0.003	Ciliophora	Intramacronucleata	Conthreep	unclass.	
Otu11	305	130	185	418	322	445	0.02	Mucoromycota	Incertae_Sedis	Mortierellales	Mortierellaceae	Mortierella
Otu27	18	21	20	302	116	441	0.001	Ascomycota	Eurotiomycetes	Eurotiales	uncultured	uncultured_ge
Otu17	15	0	0	10	446	797	0.039	Basidiomycota	Agaricomycetes	Corticiales	Corticiaceae	Sistotrema
Otu23	312	113	237	863	378	741	0.002	Cercozoa	Thecofilosea	Cryomonadida	Rhizaspididae	Rhogostoma
Otu5	0	0	0	1447	1186	42	0.001	Apicomplexa	Conoidasida	Gregarinasina	Neogregarinorida	Neogregarinorida_ge

B. The most abundant OTUs significatly separating the varieties Kariera (K) and Agria (A) (Metastats, $p < 0.05$)

Group	LB	LK	LA	HB	HK	HA	p-value	Phylum	Class	Order	Family	Genus
Otu63	1	341	0	1	2	0	0.03	Arthropoda	Arachnida	Acari_or	Acari_fa	Acari_ge
Otu94	0	0	0	0	311	0	0.001	Basidiomycota	Agaricomycetes	Agaricales	unclass.	
Otu116	0	0	0	0	231	0	0.001	Basidiomycota	Agaricomycetes	Agaricales	Marasmiaceae	Marasmius
Otu104	290	54	26	8	203	14	0.023	Basidiomycota	Malasseziomycetes	Malasseziales	Malasseziaceae	Malassezia
Otu178	50	60	15	7	72	23	0.004	Ascomycota	Sordariomycetes	Hypocreales	unclass.	
Otu288	0	0	0	10	76	0	0.001	Blastocladiomycota	Blastocladiomycetes	Blastocladiales	Blastocladiaceae	Allomyces
Otu196	46	61	13	3	50	25	0.005	Ascomycota	Sordariomycetes	Sordariales	Chaetomiaceae	Chaetomium
Otu286	4	6	1	5	51	2	0.031	Ascomycota	Pezizomycetes	Pezizales	Ascodesmidaceae	uncultured
Otu400	0	47	0	0	0	0	0.001	Mucoromycota	Glomeromycetes	Diversisporales	unclass.	
Otu578	0	0	0	0	36	0	0.001	Basidiomycota	Agaricomycetes	Agaricales	Agaricales_fa	Agaricales_ge
Otu397	0	26	0	2	8	1	0.015	unclass.				
Otu466	6	19	0	1	4	1	0.018	unclass.				
Otu599	0	22	0	0	0	0	0.001	unclass.				
Otu738	0	0	0	0	15	0	0.001	unclass.				
Otu366	8	14	5	4	10	4	0.046	Chlorophyta_ph	Trebouxiophyceae	Incertae_Sedis	Incertae_Sedis_fa	Coccomyxa
Otu764	2	13	0	0	0	0	0.001	unclass.				
Otu3556	0	12	0	0	0	0	0.001	Arthropoda	unclass.			
Otu953	0	0	0	0	11	0	0.001	unclass.				
Otu779	1	8	1	0	4	0	0.006	Chytridiomycota	Incertae_Sedis	unclass.		
Otu1075	2	4	0	0	7	0	0.001	Peronosporomycetes	Peronosporomycetes_cl	Peronosporomycetes_or	Peronosporomycetes_fa	uncultured
Otu710	0	0	0	0	10	0	0.001	Ciliophora	Intramacronucleata	Spirotrichea	Hypotrichia	unclass.
Otu2032	0	0	0	35	9	0	0.001	Ciliophora	Intramacronucleata	Spirotrichea	Hypotrichia	unclass.
Otu905	0	8	0	0	0	0	0.001	Arthropoda	unclass.			
Otu729	2	9	2	0	1	0	0.048	unclass.				
Otu1051	0	8	0	0	0	0	0.001	Euglenozoa	Kinetoplastea	Metakinetoplastina	Trypanosomatida	unclass.
Otu1780	2	6	2	1	7	4	0.046	Ascomycota	Sordariomycetes	Hypocreales	Bionectriaceae	Clonostachys
Otu1158	0	2	0	0	5	0	0.001	unclass.				
Otu1303	2	4	0	0	2	0	0.001	Ascomycota	Sordariomycetes	Hypocreales	Cordycipitaceae	unclass.
Otu1044	0	6	0	0	0	0	0.001	unclass.				
Otu2975	0	0	0	0	5	0	0.001	unclass.				

Group	LB	LK	LA	HB	HK	HA	p-value	Phylum	Class	Order	Family	Genus
Otu167	19	15	25	19	3	15	0.045	Chlorophyta_ph	Trebouxiophyceae	unclass.		
Otu277	19	4	25	1	5	5	0.036	Ascomycota	Leotiomycetes	Helotiales	Sclerotiniaceae	Sclerotinia
Otu241	19	11	21	7	0	11	0.022	unclass.				
Otu202	12	14	15	13	2	23	0.028	Chlorophyta_ph	Chlorophyceae	Chlorophyceae_or	Chlorophyceae_fa	Chlorophyceae_ge
Otu355	17	7	9	26	2	22	0.027	Cercozoa	Glissomonadida	Glissomonadida_or	Glissomonadida_fa	Glissomonadida_ge
Otu181	19	14	15	37	2	24	0.05	Cercozoa	Cercomonadidae	Cercomonadidae_or	Cercomonadidae_fa	Cercomonas
Otu249	19	7	21	6	3	11	0.019	unclass.				
Otu306	6	0	14	4	4	14	0.003	Cercozoa	unclass.			
Otu404	11	7	12	13	1	22	0.001	unclass.				
Otu429	0	0	0	0	0	27	0.001	Nematoda	Enoplea	Dorylaimia_or	Dorylaimida_fa	Dorylaimida_ge
Otu323	9	2	10	14	8	28	0.05	Schizoplasmodiida	Schizoplasmodiida_cl	Schizoplasmodiida_or	Schizoplasmodiida_fa	Ceratiomyxella
Otu3439	5	0	28	0	0	0	0.001	Ascomycota	Leotiomycetes	unclass.		
Otu248	20	0	7	7	4	25	0.023	Ciliophora	Intramacronucleata	Spirotrichea	Hypotrichia	uncultured
Otu163	12	9	28	34	15	26	0.041	Cercozoa	unclass.			
Otu216	30	11	22	33	6	26	0.009	Chlorophyta_ph	Trebouxiophyceae	Incertae_Sedis		
Otu166	22	4	14	32	5	26	0.031	Cercozoa	Cercomonadidae	Cercomonadidae_or	Cercomonadidae_fa	Cercomonas
Otu2943	28	8	20	17	4	22	0.036	Chlorophyta_ph	Chlorophyceae	Chlorophyceae_or	Chlorophyceae_fa	Chlorophyceae_ge
Otu588	8	0	10	1	0	22	0.003	Zoopagomycota	Basidiobolomycetes	Basidiobolales	Basidiobolaceae	Basidiobolus
Otu141	20	21	40	4	17	34	0.014	Cercozoa	Glissomonadida	Glissomonadida_or	Glissomonadida_fa	Glissomonadida_ge
Otu118	88	35	40	108	17	49	0.048	Chlorophyta_ph	Chlorophyceae	Chlorophyceae_or	Chlorophyceae_fa	Chlorophyceae_ge
Otu191	2	16	38	6	3	24	0.007	unclass.				
Otu90	63	26	47	61	32	61	0.04	unclass.				
Otu168	59	23	40	39	8	42	0.043	Cercozoa	unclass.			
Otu158	74	9	19	31	19	60	0.035	Cercozoa	Cercomonadidae	Cercomonadidae_or	Cercomonadidae_fa	Cercomonas
Otu61	76	71	93	87	42	90	0.026	Ascomycota	Dothideomycetes	Capnodiales	Extremaceae	Extremus
Otu78	38	34	56	38	39	105	0.005	Cercozoa	Cercomonadidae	Cercomonadidae_or	Cercomonadidae_fa	Paracercomonas
Otu133	0	0	126	0	0	0	0.001	Arthropoda	Ellipura	Collembola	Collembola_fa	Collembola_ge
Otu13	730	401	459	565	364	468	0.029	Basidiomycota	Tremellomycetes	unclass.		
Otu255	26	32	171	19	15	50	0.007	Cercozoa	Glissomonadida	Glissomonadida_or	Glissomonadida_fa	Glissomonadida_ge
Otu21	243	136	226	168	123	215	0.001	Ascomycota	Leotiomycetes	Helotiales	Incertae_Sedis	Tetracladium

Supplementary Table S11. Comparison of studies related to the topic.

**Publication**	Rozenzweig et al. 2012	Kobayashi et al. 2015	Sagova-Mareckova et al. 2015	Tomihava et al. 2017	Shi et al. 2019	Kopecky et al. 2019
**System description: treatments, sampling**	2 fields: induced suppressive and conducive soil, 1 cultivar, 1 compartment	1 field: infested artificially infested, 8 cultivars, 3 compartments	4 fields: 2 naturally suppressive and 2 conducive soils, 3 cultivars	1 field: 1 cultivar and 3 treatments, 3 compartments	1 field: soil patchiness and potato cultivar genotypes (no treatments)	2 fields: naturally suppressive and conducive soil
Actinobacteria* / *Streptomycetaceae	Similar proportion in CS/SS soils		*Actinobacteria* quantity same or high in SS	High actinobacteria in low scab, antagonistic isolates	No differences in Streptomyces	*Actinobacteria* quantity low in SS, *Streptomyces* proportion high in SS
**Pathogen / *txt* genes**	cultivation/leisons, higher percentage in conducive soil	severity/qPCR txtAB, differences between cultivars in tubers, not rhizosphere	severity/qPCR txtB, differences between tuberoshere (both sites), bulk and periderm (one site)	severity/qPCR txtAB, differences between treatments in tubers	severity/qPCR txtAB, low s in low scab in geocaulosphere	severity / qPCR *txtB*, differences between cultivars in tuberosphere
**Suppressive soil / low scab**	Bacteria diversity high *Acidobacteria, Nocardioidaceae, Pseudomonadaceae, Lysobacter, Rhizobium*	NA	Bacteria quantity similar	Actinobacteria, Streptomyces	Bacteria diversity high, quantity low, network interactions high	Bacteria quantity low. *Acetobacteraceae, Paenibacillaceae, Pseudomonadaceae, Bradyrhizobiaceae*
**Conducive soil / high scab**	Bacteria diversity low *Deinococcus-Thermus, Firmicutes, Acetobacteraceae, Bacillaceae, Trupera*	NA	NA			
**Cultivars**	Snowden - moderately resistant	resistant: Yukirasha, 02005-10, moderately resistant: Snow March, Star Ruby, Snowden, susceptible: Irish Cobbler, Toyoshiro, Piruka	susceptible: Agria, David, Valfi	Nishiyutaka	Favorita - susceptible	Kariera - resistant; Agria - susceptible

**Bacteria: cultivar - resistant / low scab**	NA	*Gammaproteobacteria Aquicella siphonis*	NA	NA	*Acidobacteria, Actinobacteria, Firmicutes, Geodermatophilaceae, Nocarioides, Curtobacterium*	Bacterial diversity high, network interactions high. *Streptomycetaceae, Rhizobiales, Sphingobacteraceae*
**Bacteria: cultivar - susceptible / high scab**	NA	*Rhodococcus, Streptomyces*	NA	NA	*Proteobacteria, Bacteroidetes, Stenotrophomonas, Variovorax, Sphingobium, Agrobacterium*	low bacterial but high archaeal and microeukaryot diversity. *Gaiellales, Micrococcales, Frankiales and Streptomycetales*
**Soil compartment effect**	NA	rhizosphere	tuberosphere, periderm	rhizoshere, tuber	geocaulosphere	tuberosphere, bulk
**Nutrients - low scab**	NA	NA	high Mg	OM	low N-NH_4, TC, OM	soil S, periderm Mg, Fe
**Nutrients - high scab**	NA	NA	high pH, C, N, Ca, Fe (total)			soil N, C, P, Ca
**Functions**	antibiosis of isolates (Meng et al. 2012)			antibiosis of isolates	high nitrogen metabolism, drug metabolism in high scab, high carbohydrate metabolism, energy metabolism, antibiotic pathways, more complex networks in low scab	

Supplementary Figure S1. Taxonomic composition of tuberosphere and bulk soil bacterial communities.

A. Relative proportions of orders within the phylum *Actinobacteria* [%].

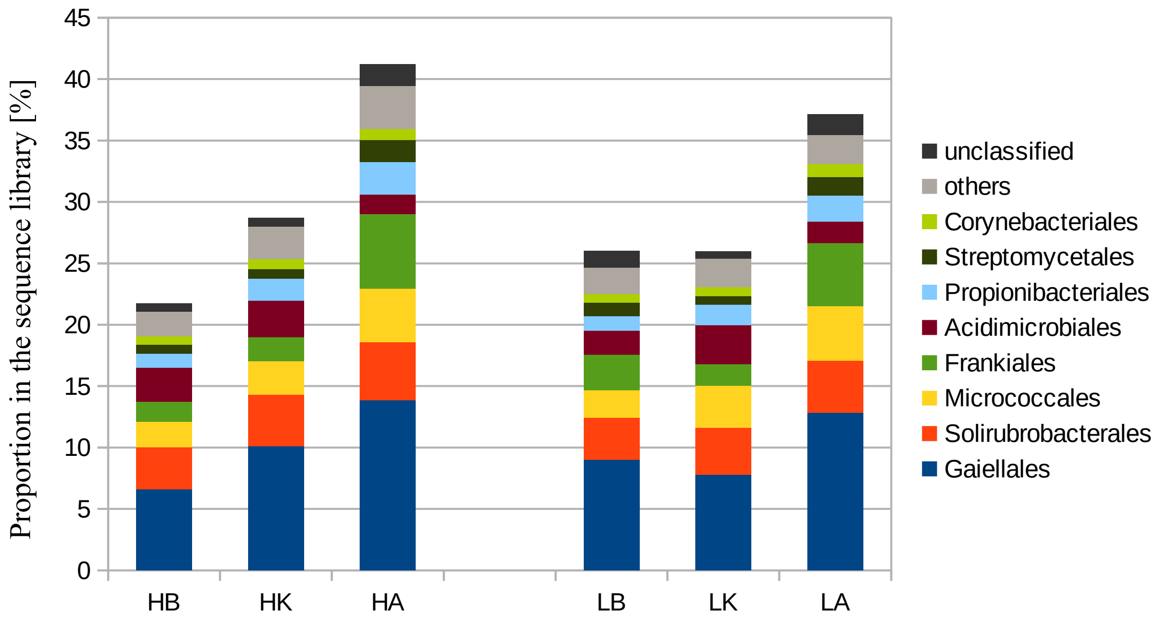

B. Relative proportions of families within the phylum *Firmicutes* [%].

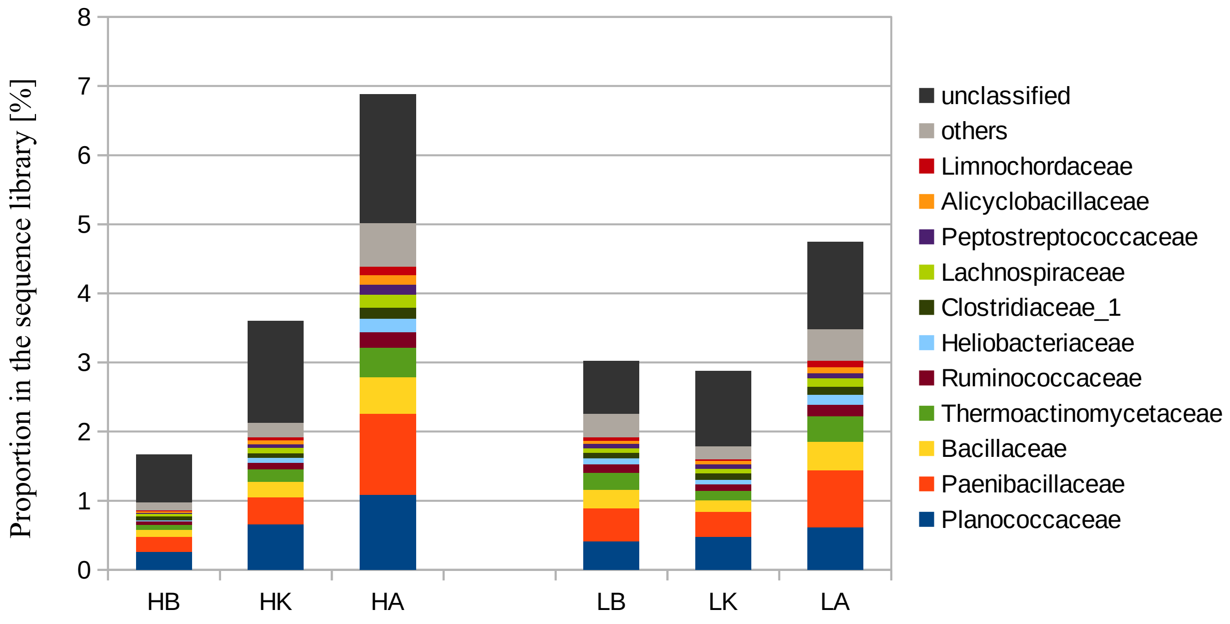

C. Relative proportions of families within the phylum *Bacteroidetes* [%].

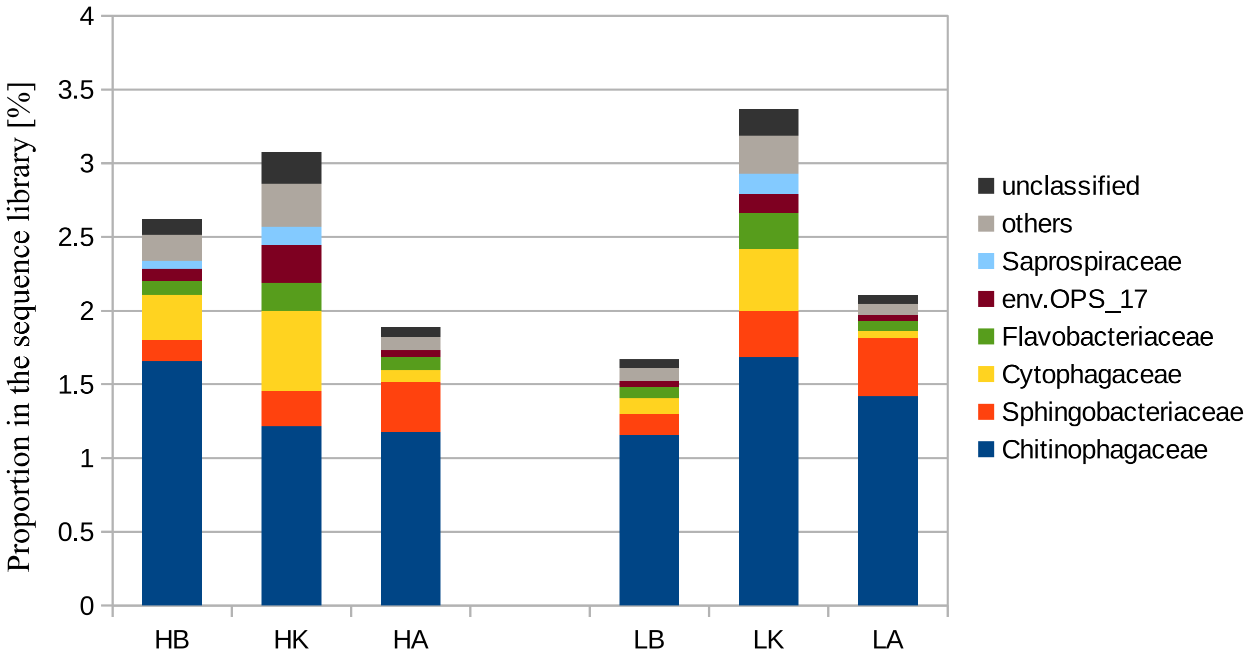

Supplementary Figure S2. Rarefaction analysis of sequence libraries of bacteria (A), archaea (B), and eukaryotes (C) from bulk (brown) and tuberosphere soil of varieties Kariera (green) and Agria (red) from suppressive field L (left) and conducive field H (right).

A. Bacteria

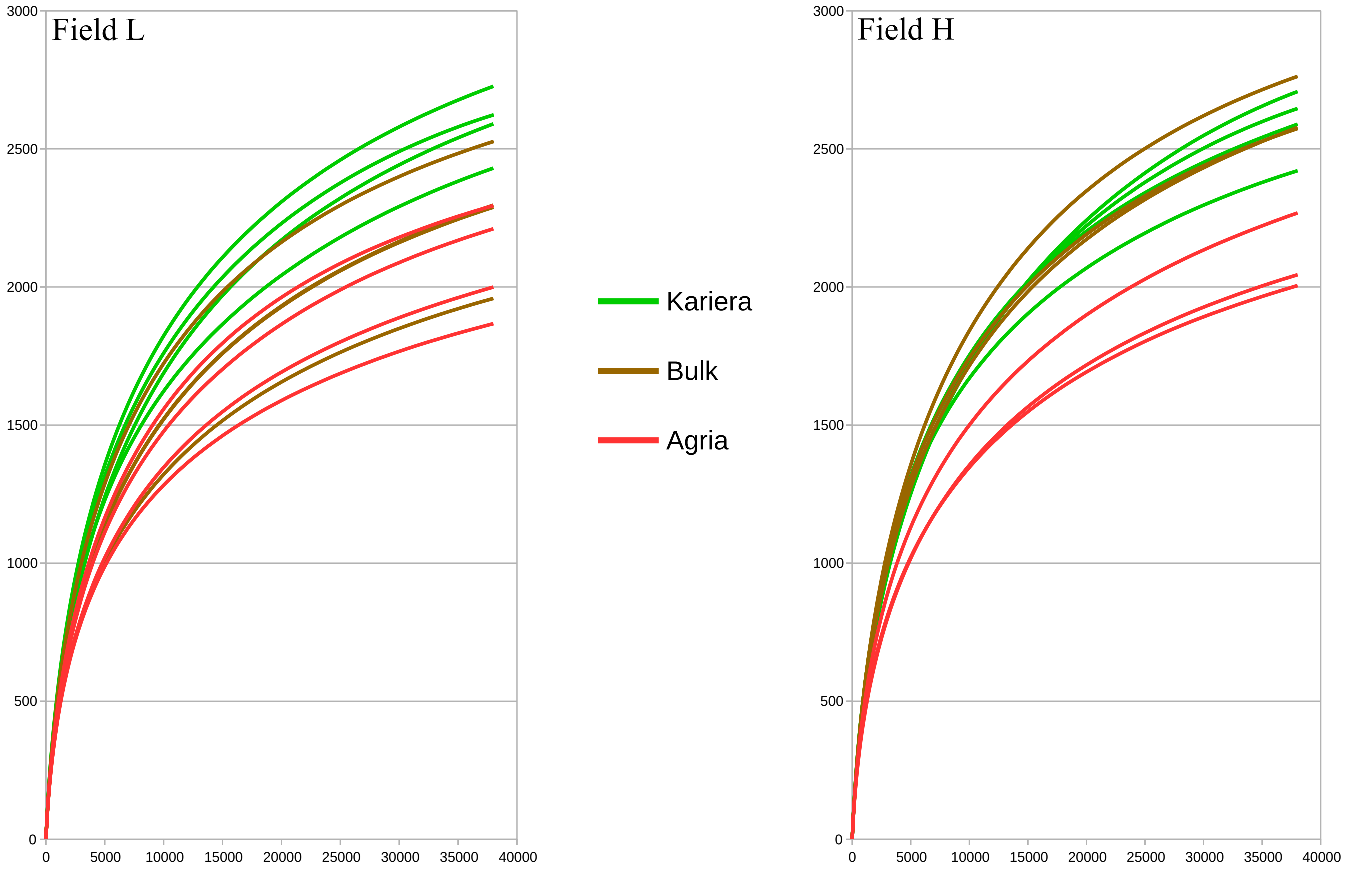

B. Archaea

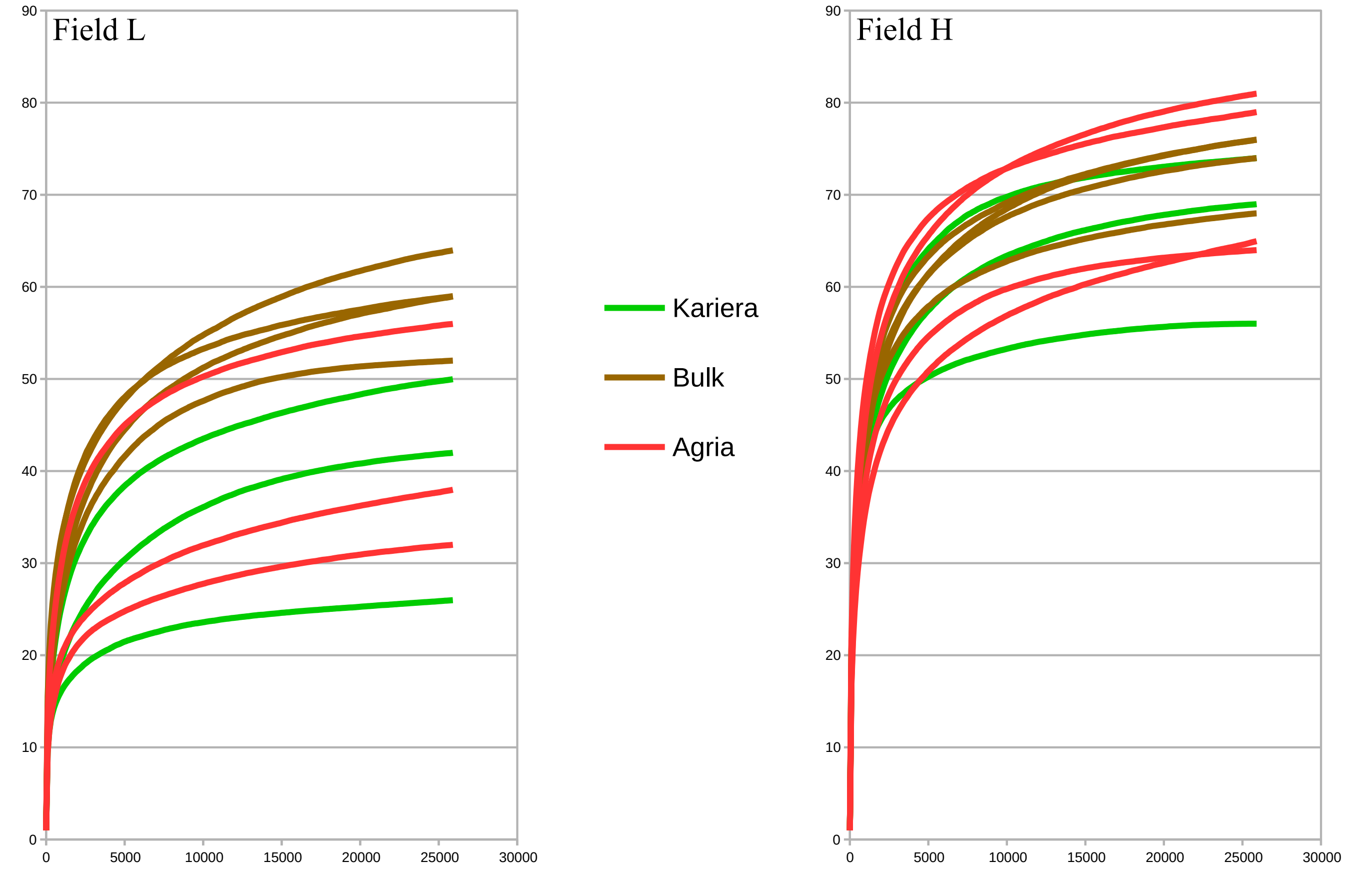

C. Eukaryotes

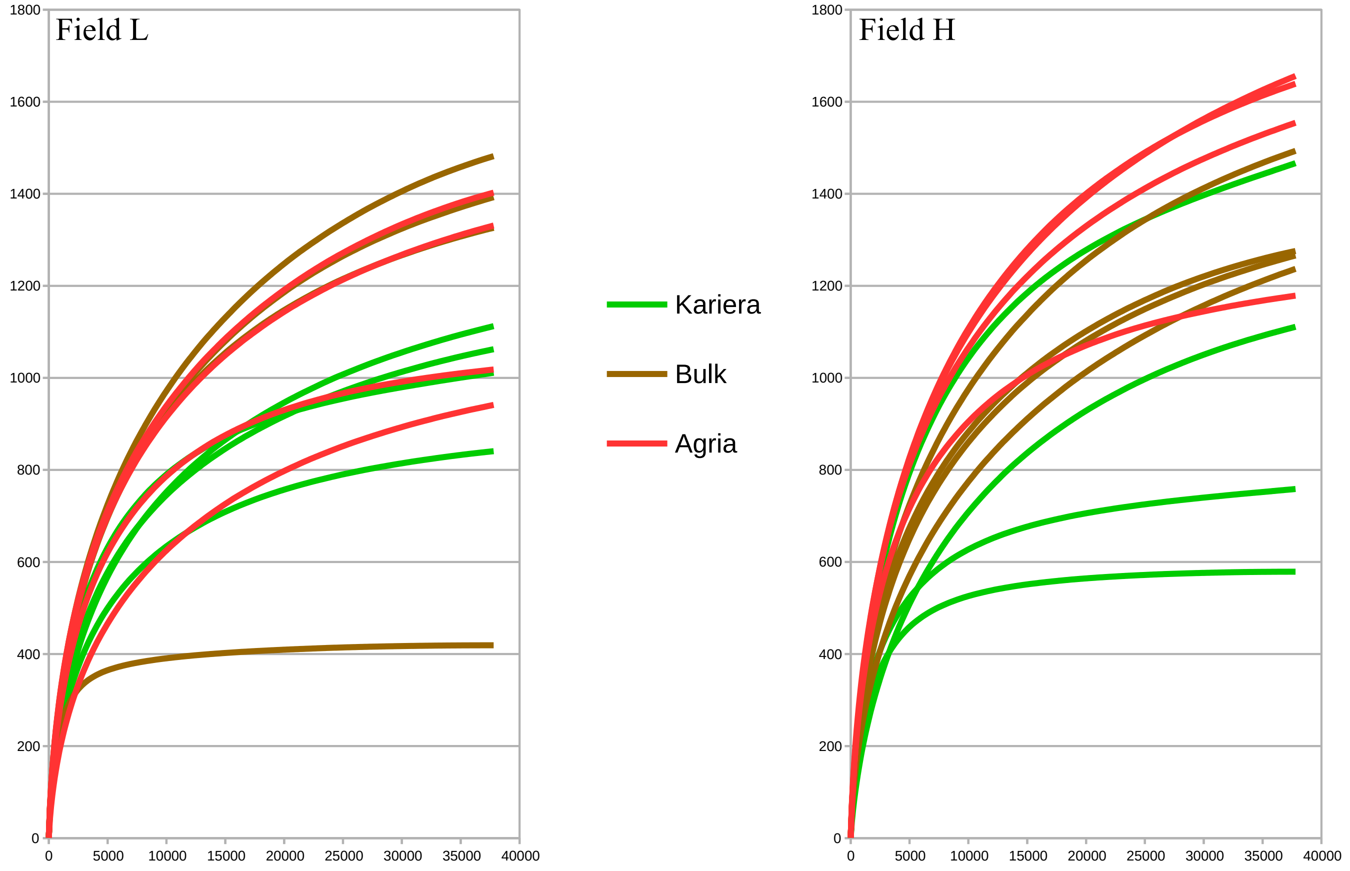

Supplementary Figure S3. Taxonomic composition of tuberosphere and bulk soil archaeal communities.

Proportions of orders within the domain *Archaea*.

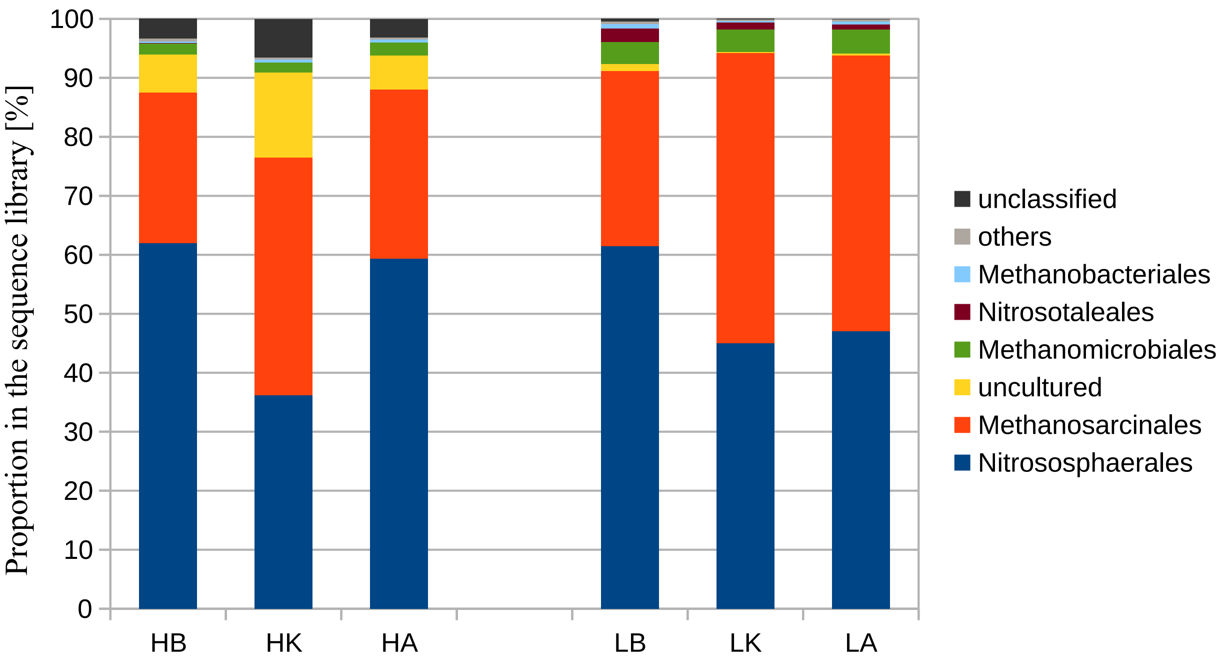

Supplementary Figure S4. Taxonomic composition of tuberosphere and bulk soil communities of micro-eukaryotes.

A. Taxonomic composition of the community of Ascomycota.

Proportions of classes within the phylum Ascomycota.

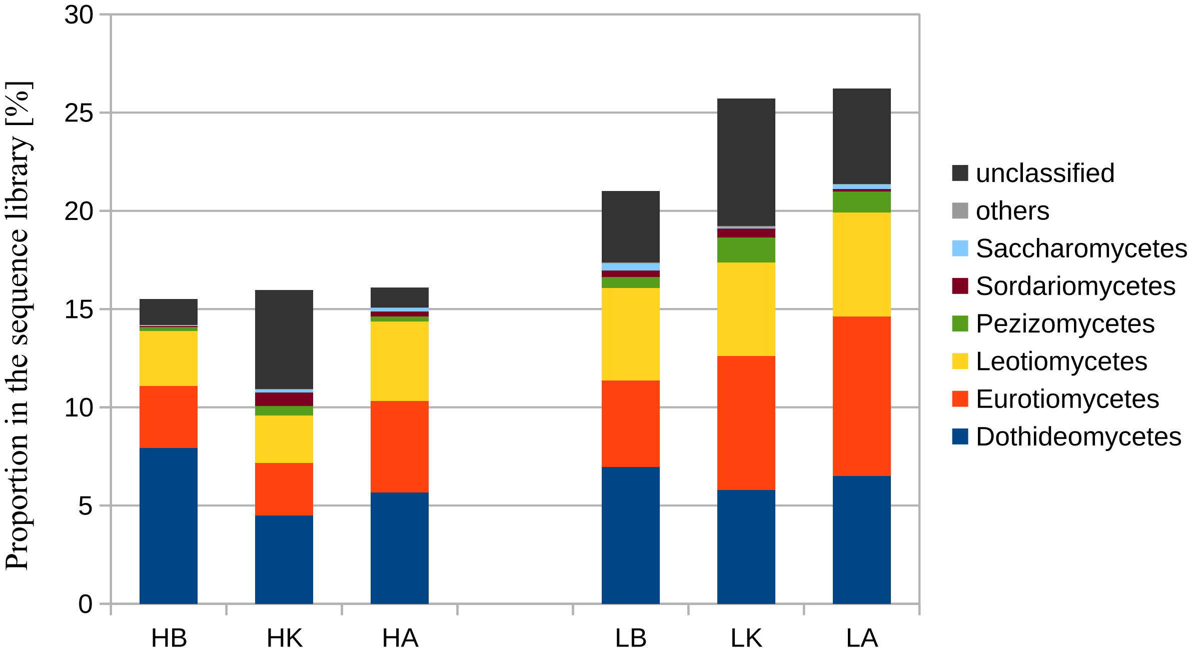

Proportions of orders within the phylum Ascomycota.

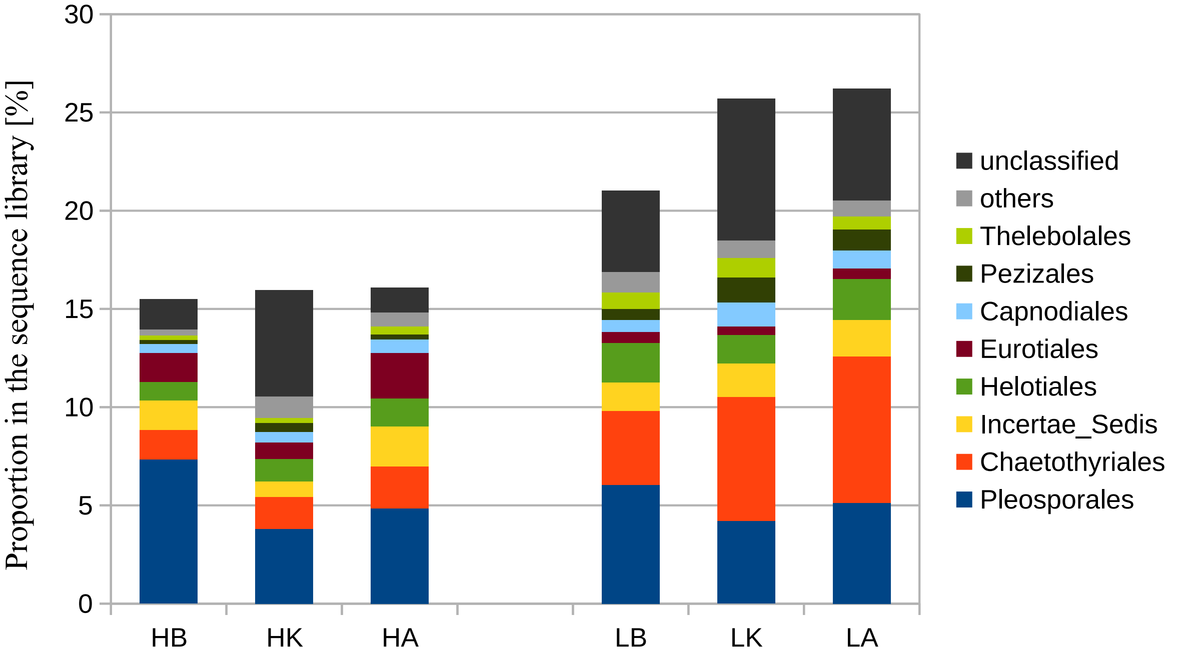

B. Proportions of taxonomic groups (classes and above) within the community of Ciliophora

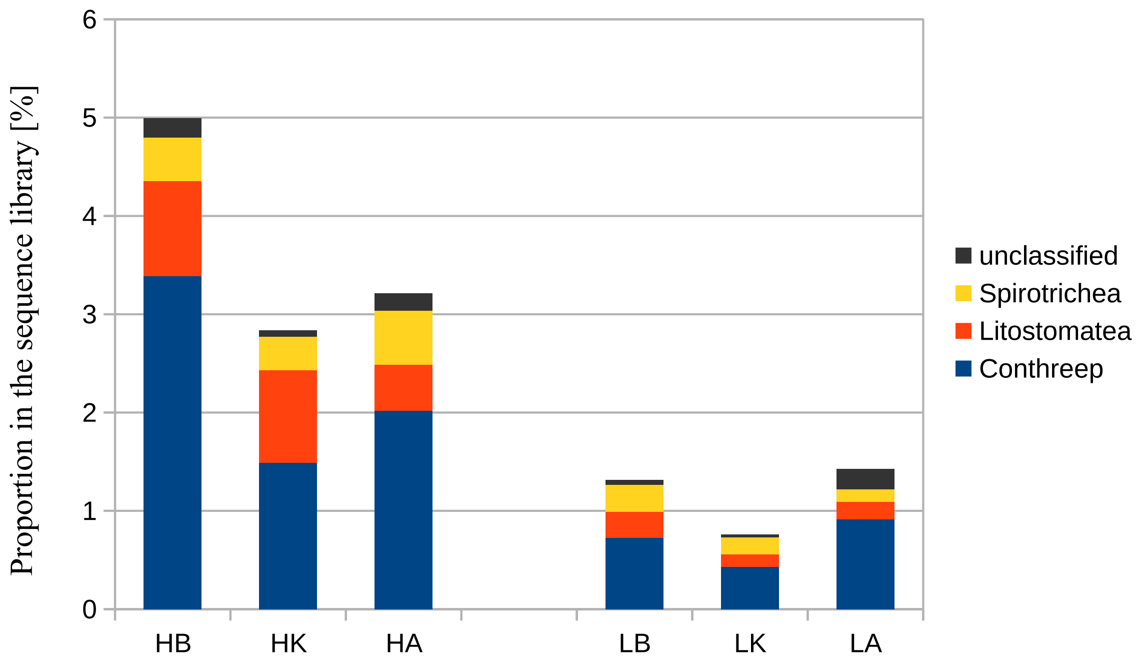

Supplement: Supplementary file 1 — Supplementary file [file 41598_2019_51570_MOESM1_ESM.pdf]
